# White blood cell count is not associated with flow-mediated vasodilation or nitroglycerine-induced vasodilation

**DOI:** 10.1038/s41598-022-12205-5

**Published:** 2022-05-17

**Authors:** Shinji Kishimoto, Tatsuya Maruhashi, Masato Kajikawa, Takahiro Harada, Takayuki Yamaji, Yiming Han, Aya Mizobuchi, Yu Hashimoto, Kenichi Yoshimura, Yukiko Nakano, Kazuaki Chayama, Chikara Goto, Farina Mohamad Yusoff, Ayumu Nakashima, Yukihito Higashi

**Affiliations:** 1 Department of Cardiovascular Regeneration and Medicine, Research Institute for Radiation Biology and Medicine, Hiroshima University, 1-2-3 Kasumi, Minami-ku, Hiroshima, 734-8551 Japan; 2 Division of Regeneration and Medicine, Medical Center for Translational and Clinical Research, Hiroshima University Hospital, Hiroshima, Japan; 3 Department of Cardiovascular Medicine, Graduate School of Biomedical and Health Sciences, Hiroshima University, Hiroshima, Japan; 4 Department of Gastroenterology and Metabolism, Graduate School of Biomedical and Health Sciences, Hiroshima University, Hiroshima, Japan; 5 Dpartment of Rehabilitation, Faculty of General Rehabilitation, Hiroshima International University, Hiroshima, Japan; 6 Department of Stem Cell Biology and Medicine, Graduate School of Biomedical and Health Sciences, Hiroshima University, Hiroshima, Japan

**Keywords:** Biomarkers, Cardiology

## Abstract

It is well known that white blood cell (WBC) count is an independent predictor of cardiovascular events. However, associations of WBC count and WBC subtypes with endothelial function assessed by flow-mediated vasodilation (FMD) and vascular smooth muscle function assessed by nitroglycerine-induced vasodilation (NID) are unclear. The aim of this study was to determine the relationships of WBC count and WBC subtypes with vascular function assessed by FMD and NID. A total of 1351 subjects in whom FMD and NID had been measured were recruited from Hiroshima University Vascular Registry. Mean values were 3.7 ± 2.8% for FMD and 11.8 ± 5.9% for NID. WBC was not correlated with FMD or NID. NID was significantly correlated with lymphocytes in univariate analysis but not with other hematologic parameters. In multiple linear regression analyses, NID was not correlated with lymphocytes. In all subgroups including subgroups of age, gender, body mass index, hypertension, dyslipidemia, diabetes mellitus, smoking and tertile of WBC count, WBC count was not correlated with FMD or NID. WBC count and WBC subtypes were not associated with endothelial function assessed by FMD or vascular smooth muscle function assessed by NID. WBC count and vascular function assessed by FMD and NID may reflect different aspects of atherosclerosis.

**Clinical Trial Registration Information:** URL for Clinical Trial: http://www.umin.ac.jp Registration Number for Clinical Trial: UMIN000039512.

## Introduction

Inflammation plays an critical role in the development of atherosclerosis, inducing cardiovascular complications^1^. Measurement of white blood cell (WBC) count is inexpensive and WBC count is widely used as a simple biomarker of systemic inflammation. Several studies have shown that there is a positive correlation between WBC count and risk of coronary heart disease (CHD) and that WBC count is an independent predictor of cardiovascular events^2–4^. In addition, WBC subtypes including neutrophils, monocytes, lymphocytes and eosinophils have been shown to be correlated with the risk of CHD^5,6^. Monocytes that have been supplied from the peripheral blood into the vessel wall differentiate into macrophages that phagocytose lipids in an atherosclerotic lesion^7^. Neutrophils accumulate in the plaque. An atherosclerotic lesion in which supplement and reservoir of inflammatory cells increase leads to cardiovascular events^8^. Endothelial dysfunction is well known to be the initial step in the development of atherosclerosis^1,9^. However, there is little information on the relationship of WBC count with endothelial function and there is no information on the relationship of WBC count with vascular smooth muscle function.

Flow-mediated vasodilation (FMD) and nitroglycerine-induced vasodilation (NID) have been widely used^10,11^. FMD and NID assess endothelial function reflected in nitric oxide (NO) production and vascular smooth muscle function, respectively. Several investigators have shown that FMD and NID were associated with risk factors of atherosclerosis and were independent predictors of cardiovascular events^12–14^.

Associations of WBC count and WBC subtypes with endothelial function assessed by FMD and vascular smooth muscle function assessed by NID are unclear. The aim of this study was to determine the relationships of WBC count and WBC subtypes with vascular function assessed by FMD and NID.

## Results

### Baseline clinical characteristics

The baseline clinical characteristics of the subjects are summarized in Table 1. Of the 1351 subjects, 1051 (77.8%) had hypertension, 853 (63.1%) had dyslipidemia, 444 (32.9%) had diabetes mellitus, 210 (15.5%) had previous coronary heart disease, 94 (7.0%) had previous stroke, and 229 (17.0%) were current smokers. Mean values were 3.7 ± 2.8% for FMD and 11.8 ± 5.9% for NID. Mean values were 5.91 ± 1.43 × 10^3^/μL for WBC, 3.51 ± 1.10 × 10^3^/μL for neutrophils, 1.83 ± 0.59 × 10^3^/μL for lymphocytes, 0.34 ± 0.12 × 10^3^/μL for monocytes, 0.16 ± 0.12 × 10^3^/μL for eosinophils and 0.03 ± 0.02 × 10^3^/μL for basophils.

**Table 1:** Clinical characteristics of the subjects.

Variables	Total (n = 1351)
Age, year	63 ± 14
Men, n (%)	812 (60.1)
Body mass index, kg/m^2^	24.2 ± 4.2
Systolic blood pressure, mmHg	133 ± 19
Diastolic blood pressure, mmHg	79 ± 12
Heart rate, bpm	71 ± 12
Total cholesterol, mmol/L	4.99 ± 0.98
Triglycerides, mmol/L	1.58 ± 1.05
HDL cholesterol, mmol/L	1.53 ± 0.44
LDL cholesterol, mmol/L	2.92 ± 0.88
Glucose, mmol/L	6.61 ± 2.39
Hemoglobin A1c, %	5.8 ± 0.9
BUN, mmol/L	5.71 ± 1.89
Creatinine, umol/L	73.4 ± 25.6
eGFR, mL/min/1.73 m^2^	71 ± 20
hs-CRP, mg/dL	0.16 ± 0.34
**Medical history, n (%)**	
Hypertension	1051 (77.8)
Dyslipidemia	853 (63.1)
Diabetes mellitus	444 (32.9)
Previous coronary heart disease	210 (15.5)
Previous stroke	94 (7.0)
Current smoker, n (%)	229 (17.0)
**Medication, n (%)**	
Antiplatelets	314 (23.2)
Calcium channel blockers	641 (47.4)
ACEIs or ARBs	511 (37.8)
β-blockers	282 (20.9)
Diuretics	161 (11.9)
Statins	500 (37.0)
**Medically treated diabetes mellitus**	
Any	307 (22.7)
Insulin dependent	35 (2.6)
Baseline BAD, mm	4.1 ± 0.7
FMD, %	3.7 ± 2.8
NID, %	11.8 ± 5.9

Results are presented as means ± SD for continuous variables and percentages for categorical variables.

*HDL* high-density lipoprotein, *LDL* low-density lipoprotein, *BUN* blood urea nitrogen, *eGFR* estimated-glomerular filtration rate, *hs-CRP* high-sensitive C-reactive protein, *ACEIs* angiotensin-converting enzyme inhibitors, *ARBs* angiotensin II receptor blockers, *BAD* brachial artery diameter, *FMD* flow-mediated vasodilation, *NID* nitroglycerine-induced vasodilation.

The baseline characteristics in subgroups including subgroups of age, gender, BMI, hypertension, dyslipidemia, diabetes mellitus, smoking, tertile of WBC count and baseline brachial artery diameter (BAD) are summarized in Supplemental Tables S1–S10.

### Relationships of WBC and vascular function with variables

The univariate relations between WBC, FMD, NID and variables are shown in Table 2. WBC count was positively correlated with body mass index (BMI), diastolic blood pressure, heart rate, total cholesterol, triglycerides, low-density lipoprotein (LDL) cholesterol, hemoglobin A1c, creatinine, high-sensitive C-reactive protein (hs-CRP), smoking and baseline BAD and was negatively correlated with age and high-density lipoprotein (HDL) cholesterol. Other parameters were not correlated with WBC count. FMD was negatively correlated with age, systolic blood pressure, triglycerides, glucose, hemoglobin A1c, blood urea nitrogen (BUN), creatinine, estimated-glomerular filtration rate (eGFR), smoking and baseline BAD. Other parameters were not correlated with FMD. NID was positively correlated with total cholesterol and was negatively correlated with age, systolic blood pressure, hemoglobin A1c, BUN, creatinine, eGFR, smoking and baseline BAD. Other parameters were not correlated with NID.

**Table 2:** Univariate analysis of relationships among white blood cell, FMD, NID and variables.

Variables	White blood cell	FMD	NID
Age, year	− 0.13^†^	− 0.25^†^	− 0.28^†^
Body mass index, kg/m^2^	0.21^†^	− 0.01	− 0.01
Systolic blood pressure, mmHg	0.06	− 0.10^†^	− 0.10^†^
Diastolic blood pressure, mmHg	0.09^†^	− 0.02	0.06
Heart rate, bpm	0.08^†^	0.02	0.01
Total cholesterol, mmol/L	0.08^†^	− 0.02	0.10^†^
Triglycerides, mmol/L	0.20^†^	− 0.07*	0.05
HDL cholesterol, mmol/L	− 0.21^†^	− 0.01	0.01
LDL cholesterol, mmol/L	0.11^†^	− 0.01	0.06
Glucose, mmol/L	0.04	− 0.14^†^	− 0.03
Hemoglobin A1c, %	0.13^†^	− 0.13^†^	− 0.09^†^
BUN, mmol/L	− 0.01	− 0.08^†^	− 0.14^†^
Creatinine, umol/L	0.13^†^	− 0.08^†^	− 0.10^†^
eGFR, mL/min/1.73 m^2^	0.02	0.14^†^	0.16^†^
hs-CRP, mg/dL	0.16^†^	0.06	− 0.02
Smoking, pack-years	0.11^†^	− 0.08*	− 0.09*
White blood cells, × 10^3^/μL	–	− 0.01	0.03
Neutrophils, × 10^3^/μL	0.88^†^	0.02	− 0.01
Lymphocytes, × 10^3^/μL	0.54^†^	0.01	0.08*
Monocytes, × 10^3^/μL	0.59^†^	0.02	− 0.04
Eosinophils, × 10^3^/μL	0.28^†^	0.04	0.05
Basophils, × 10^3^/μL	0.25^†^	0.04	− 0.04
Baseline BAD, mm	0.08^†^	− 0.34^†^	− 0.40^†^
FMD, %	− 0.01	–	0.40
NID, %	0.03	0.40	–

*FMD* flow-mediated vasodilation, *NID* nitroglycerine-induced vasodilation, *HDL* high-density lipoprotein, *LDL* low-density lipoprotein, *BUN* blood urea nitrogen, *eGFR* estimated-glomerular filtration rate, *hs-CRP* high-sensitive C-reactive protein, *BAD* brachial artery diameter.

*P < 0.05, ^†^P < 0.01.

The univariate relations between WBC, FMD, NID and variables in subgroups including subgroups of age, gender, BMI, hypertension, dyslipidemia diabetes mellitus, smoking, tertile of WBC count and baseline BAD are shown in Supplemental Tables S11–S35.

### Relationships of WBC with vascular function

**Figure 1:**
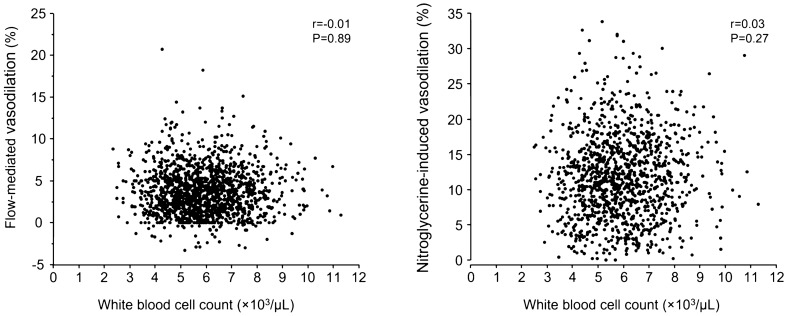
Scatter plots show the relationships between white blood cell count and flow-mediated vasodilation and nitroglycerine-induced vasodilation.

WBC was not correlated with both FMD and NID (r = − 0.01 and r = 0.03, P = 0.89 and P = 0.27, respectively; Fig. 1). In all subgroups including subgroups of age, gender, BMI, hypertension, dyslipidemia, diabetes mellitus, smoking, tertile of WBC count and baseline BAD, WBC was not correlated with FMD or NID in univariate analysis (Table 3).

**Table 3:** Univariate analysis of relationships between white blood cell, FMD and NID in subsets of subjects.

Variables	FMD	NID
< 65 years old (n = 625)	− 0.06	− 0.01
≥ 65 years old (n = 726)	0.01	− 0.01
Men (n = 812)	0.01	0.05
Women (n = 539)	0.02	0.01
Body mass index < 25 kg/m^2^ (n = 840)	0.13	0.04
Body mass index ≥ 25 kg/m^2^ (n = 501)	− 0.05	0.01
Hypertension (n = 1051)	0.01	0.01
No hypertension (n = 300)	− 0.03	0.09
Dyslipidemia (n = 853)	− 0.02	0.05
No dyslipidemia (n = 498)	0.03	0.01
Diabetes mellitus (n = 444)	0.04	0.05
No diabetes mellitus (n = 907)	− 0.01	0.04
Current smoker (n = 229)	− 0.05	0.01
Non-smoker (n = 1122)	0.01	0.02
Low WBC ≤ 5.2 × 10^3^/μL (n = 458)	− 0.01	0.06
Middle WBC 5.2–6.5 × 10^3^/μL (n = 455)	0.06	− 0.01
High WBC > 6.5 × 10^3^/μL (n = 438)	− 0.03	0.05

*FMD* flow-mediated vasodilation, *NID* nitroglycerine-induced vasodilation, *WBC* white blood cell.

Multiple linear regression analyses were performed to identify independent variables associated with FMD, NID and baseline BAD. Multiple linear regression analyses revealed that WBC count was not correlated with FMD, NID or baseline BAD (β = − 0.02, P = 0.47 for FMD, β = 0.004, P = 0.78 for NID and β =  − 0.01, P = 0.78 for baseline BAD; Supplemental Tables S36–S38).

### Relationships of WBC subtypes with vascular function

Lymphocytes were correlated with NID (r = 0.08, P = 0.02) but were not correlated with FMD (Table 2). Neutrophils, monocytes, eosinophils and basophils were not correlated with FMD or NID (Table 2). Multiple linear regression analyses revealed that lymphocytes were not correlated with NID (β = 0.06, P = 0.06; Supplemental Table S39).

In subjects under 65 years of age, FMD was negatively correlated with lymphocytes (r =  − 0.10, P = 0.02) and positively correlated with eosinophils (r = 0.10, P = 0.03; Supplemental Table S11). Other WBC subtypes were not correlated with FMD or NID. Multiple linear regression analyses revealed that FMD was positively correlated with eosinophils (β = 0.09, P = 0.03; Supplemental Tables S40), whereas there were no significant relationships between FMD and lymphocytes (β =  − 0.06, P = 0.10; Supplemental Table S41).

In subjects with 65 years of age or older, FMD was positively correlated with basophils (r = 0.10, P = 0.02) and NID was positively correlated with lymphocytes (r = 0.10, P = 0.04; Supplemental Table S12). Other WBC subtypes were not correlated with FMD or NID. Multiple linear regression analyses revealed that FMD was positively correlated with basophils (β = 0.10, P = 0.01; Supplemental Table S42), whereas there were no significant relationships between NID and lymphocytes (β = 0.04, P = 0.30; Supplemental Table S43).

In subjects under 35 years of age, NID was negatively correlated with basophils (r =  − 0.38, P < 0.01; Supplemental Table S13). Other WBC subtypes were not correlated with FMD or NID. Multiple linear regression analyses revealed that NID was negatively correlated with basophils (β =  − 0.41, P < 0.01; Supplemental Table S44).

In subjects between 55 and 64 years of age, FMD was positively correlated with eosinophils (r = 0.16, P = 0.01; Supplemental Table S16). Other WBC subtypes were not correlated with FMD or NID. Multiple linear regression analyses revealed that FMD was positively correlated with eosinophils (β = 0.14, P = 0.03; Supplemental Table S45).

In subjects between 65 and 74 years of age, NID was positively correlated with lymphocytes (r = 0.20, P < 0.01; Supplemental Table S17). Other WBC subtypes were not correlated with FMD or NID. Multiple linear regression analyses revealed that there were no significant relationships between NID and lymphocytes (β = 0.08, P = 0.12; Supplemental Table S46).

In subjects over 75 years of age, NID was negatively correlated with monocytes (r =  − 0.18, P = 0.04; Supplemental Table S18). Other WBC subtypes were not correlated with FMD or NID. Multiple linear regression analyses revealed that there were no significant relationships between NID and monocytes (β =  − 0.07, P = 0.13; Supplemental Table S47).

In subjects with BMI under 25 kg/m^2^, FMD was positively correlated with basophils (r = 0.09, P = 0.02) and NID was positively correlated with lymphocytes (r = 0.11, P < 0.01; Supplemental Table S21). Other WBC subtypes were not correlated with FMD or NID. Multiple linear regression analyses revealed that NID was positively correlated with lymphocytes (β = 0.10, P = 0.01; Supplemental Table S48), whereas there were no significant relationships between FMD and basophils (β = 0.08, P = 0.10; Supplemental Table S49).

In subjects without hypertension, NID was positively correlated with lymphocytes (r = 0.21, P < 0.01) and eosinophils (r = 0.16, P = 0.02; Supplemental Table S23). Other WBC subtypes were not correlated with FMD or NID. Multiple linear regression analyses revealed that NID was positively correlated with lymphocytes (β = 0.15, P = 0.03; supplemental Table S50), whereas there were no significant relationships between NID and eosinophils (β = 0.04, P = 0.54; Supplemental Table S51).

In subjects without diabetes mellitus, NID was positively correlated with lymphocytes (r = 0.08, P = 0.04; Supplemental Table S27). Other WBC subtypes were not correlated with FMD or NID. Multiple linear regression analyses revealed that there were no significant relationships between NID and lymphocytes (β = 0.07, P = 0.06; Supplemental Table S52).

In subjects with diabetes mellitus, FMD was positively correlated with basophils (r = 0.12, P = 0.03; Supplemental Table S28). Other WBC subtypes were not correlated with FMD or NID. Multiple linear regression analyses revealed that there were no significant relationships between NID and basophils (β = 0.13, P = 0.01; Supplemental Table S53).

In subjects with a middle WBC count, NID was positively correlated with lymphocytes (r = 0.15, P < 0.01) and negatively correlated with monocytes (r =  − 0.13, P = 0.02; Supplemental Table S32). Other WBC subtypes were not correlated with FMD or NID. Multiple linear regression analyses revealed that NID was positively correlated with lymphocytes (β = 0.11, P = 0.02; Supplemental Table S54), but there were no significant relationships between NID and monocytes (β =  − 0.04, P = 0.37; Supplemental Table S55).

In subjects with low baseline BAD, NID was positively correlated with lymphocytes (r = 0.12, P = 0.01) and eosinophils (r = 0.09, P = 0.04; Supplemental Table S34). Other WBC subtypes were not correlated with FMD or NID. Multiple linear regression analyses revealed that there were no significant relationships of NID with lymphocytes and eosinophils (β = 0.08, P = 0.06 for lymphocytes; β = 0.05, P = 0.25 for eosinophils; Supplemental Tables S56, S57).

In subjects with high baseline BAD, FMD was positively correlated with eosinophils (r = 0.11, P = 0.01; Supplemental Table S35). Other WBC subtypes were not correlated with FMD or NID. Multiple linear regression analyses revealed that there were no significant relationships of FMD with lymphocytes and eosinophils (β = 0.08, P = 0.60; Supplemental Table S58).

There were no significant relationships of FMD and NID with WBC subtypes in subgroups of 35–44 and 45–54 years of age, men, women, BMI over 25 kg/m^2^, hypertension, no dyslipidemia, dyslipidemia, non-smoker, current smoker and high and low WBC counts (Supplemental Tables S14, S15, S19, S20, S22, S24–S26, S29–S31, S33).

## Discussion

In the present study, we showed for the first time that WBC count and WBC subtypes were not correlated with FMD or NID. In addition, in all subgroups including subgroups of age, gender, BMI, hypertension, dyslipidemia, diabetes mellitus, smoking and tertile of WBC count, WBC was not correlated with FMD or NID. These findings suggest that WBC count and WBC subtypes are not associated with FMD or NID.

Previous studies have shown a positive correlation between WBC count and risk of CHD in a CHD-free population, patients with CHD, patients witµous myocardial infarction, smokers, non-smokers, patients with dyslipidemia, patients with diabetes mellitus and patients with chronic kidney disease^2,4,15,16^. Several studies have shown that WBC is an independent predictor of cardiovascular events^2–4^. Grimm et al. showed that WBC count was strongly related to risk of CHD. In addition, a decrease in WBC count of 1.0 × 10^3^/μL was associated with a decrease in the risk of CHD after adjustment for cardiovascular risk factors^17^. Folsom et al. showed that an elevated WBC count was significantly correlated with relative risk of CHD in subjects with free of CHD^4^. Kim et al. showed that WBC count was positively associated with Framingham risk score^18^. On the other hand, several studies have shown that both FMD and NID were independent predictors of cardiovascular events^14,19–21^. In a previous study, we showed that the combination of FMD and NID more correctly predicted cardiovascular events than did FMD or NID alone. In addition, both FMD and NID were shown to be negatively correlated with age, gender, systolic blood pressure, BMI, LDL cholesterol and smoking as risk factors of atherosclerosis^22–24^. In the present study, WBC count, FMD and NID were negatively correlated with age, blood pressure, hemoglobin A1c, smoking and Framingham risk score. These results are consistent with results of previous studies. However, WBC count and WBC subtypes were not correlated with either FMD or NID.

Some studies showed a relationship between WBC count and endothelial function. Elkind et al. showed that increasing WBC count was associated with decreasing FMD after adjusting for atherosclerosis risk factors in a population-based cohort study in which study participants were aged ≥ 40 years^25^. In addition, it was shown in that study that C. pneumoniae IgA titers in women were associated with mean decrease in FMD^25^. In the present study, we showed that WBC count was not correlated with either FMD or NID. The discrepancy between the results of previous studies and the results of our study regarding the relationship of WBC count with FMD is the cause of the different characteristics of the subjects. We enrolled the participants in present study from a general population and ≥ 18 year old age, while we excluded patients with advanced cancer, patients with infection, patients with hematologic disease, patients who had received prednisolone treatment, and patients with end-stage renal disease. Therefore, our study had a more general population than that in the previous studies since we enrolled subjects with a wider range of ages and excluded patients with endothelial dysfunction due to autoimmune disease including vasculitis and infection.

It has been shown that a low lymphocytes count is an independent predictor of CVD in patients with a high risk of cardiovascular disease and in patients with heart failure^5,26^. Phillips et al. showed that lymphocyte count in subjects over 50 years of age was positively correlated with carotid intima-media thickness (IMT) and pulse wave velocity (PWV), which were shown to be associated with risk factors of atherosclerosis and to be predictors of cardiovascular events^27^. On the other hand, some studies showed that there was no significant association of lymphocyte count with cardiovascular disease and carotid IMT and PWV in a general population^28–31^. Lymphopenia is a response to systemic stress mainly mediated by elevated levels of catecholamines and cortisol^32^. In the present study, neither FMD nor NID was not correlated with lymphocyte count in all subjects. On the other hand, NID was positively correlated with lymphocyte count in subjects with BMI under 25 kg/m^2^, in subjects without hypertension and in subjects with a middle WBC count. These findings suggest that lymphocyte count is not correlated with vascular function in the general population but is correlated with vascular function in some selective populations. It may be difficult to assess vascular function by measurement of lymphocyte count in a general population.

Measurement of WBC count is inexpensive and WBC count is widely used as a simple biomarker of systemic inflammation. Inflammation plays an important role in the development and progression of atherosclerosis^1^. Vascular inflammation caused by hypertension, dyslipidemia, diabetes mellitus, and smoking as atherosclerosis risk factors leads to endothelial dysfunction via a reduction in NO availability^33^. Stimulation of endothelial cell receptors by inflammatory cytokines leads to upregulation of adhesion molecules including vascular cell adhesion molecule-1, intercellular adhesion molecule-1, E-Selection and P-Selection. Monocytes and leukocytes adhere to endothelial cells and transmigrate into subendothelial layers. Continued vascular inflammation results in increased numbers of leukocytes and macrophages, which induce further vascular damage^34^. In the present study, endothelial dysfunction assessed by FMD and vascular smooth muscle function assessed by NID were not correlated with WBC count or WBC subtypes. Neither FMD nor NID could detect vascular inflammation as reflected in WBC count and WBC subtypes.

This study has some limitations. First, we excluded patients who had received prednisolone treatment since it is well known that prednisolone increases the number of WBCs and alters WBC phenotypes. It is thought that patients with collagen disease or vasculitis were excluded from analyses. Therefore, our results cannot be applied to those patients. Further studies are needed to confirm the relationship between WBC count and vascular function in patients with collagen disease or vasculitis. Second, baseline BAD is considered to be an important determinant of FMD and NID since FMD and NID are calculated as a percentage change in the BAD. After adjustment for the baseline BAD and cardiovascular risk factors, WBC count and WBC subtypes were not correlated with either FMD or NID. Third, multiple linear regression analyses of the relationship of FMD with eosinophils in subjects under 65 years of age showed a weak correlation despite significant P values because the present study had a large sample size. Considering that the effect sizes were small, in subjects under 65 years of age, it is possible that the relationships of vascular function with WBC subtypes are not very important in a clinical context. Further studies are needed to reconfirm the relationships between WBC subtypes and vascular function.

## Conclusion

WBC count and WBC subtypes were not associated with FMD or NID. WBC count and vascular function assessed by FMD and NID may reflect different aspects of atherosclerosis.

## Methods

### Subjects

Between April 2010 and August 2018, a total of 1713 subjects in whom FMD and NID had been measured were recruited from Hiroshima University Vascular Registry. Three hundred sixty-two of the 1713 subjects, including 123 patients with infection, 150 patients with advanced cancer, 46 patients with end-stage renal disease, 31 patients who had received prednisolone treatment, and 12 patients with hematologic disease, were excluded. Finally, 1351 subjects were enrolled in this study. Hypertension was defined as treatment with oral antihypertensive drugs or systolic blood pressure of more than 140 mm Hg or diastolic blood pressure of more than 90 mm Hg in a sitting position, on at least three different occasions. Diabetes mellitus was defined according to the American Diabetes Association or a previous diagnosis of diabetes^35,36^. Dyslipidemia was defined according to the third report of the National Cholesterol Education Program^37^.

All methods were carried out in accordance with relevant guidelines and regulations. The Ethics Review Board of Hiroshima University approved the study protocol. Written informed consent for participation in the study was obtained from all of the subjects.

### Study protocol

We measured vascular responses to reactive hyperemia and sublingual administration of nitroglycerine. Subjects fasted the previous night for at least 12 h. The study began at 8:30 AM. The subjects were kept in the supine position in a quiet, dark, and air-conditioned room (constant temperature of 22–25 °C) throughout the study. A 23-gauge polyethylene catheter was inserted into the left deep antecubital vein to obtain blood samples. Thirty minutes after maintaining the supine position, FMD and NID were measured. The observers were blind to the form of examination. We performed measurement of FMD and NID in the same protocol as our previously reported^21^.

### Measurements of FMD and NID

Vascular response to reactive hyperemia in the brachial artery was used for assessment of endothelium-dependent FMD. A high-resolution linear artery transducer was coupled to computer-assisted analysis software (UNEXEF18G, UNEX Co, Nagoya, Japan) that used an automated edge detection system for measurement of brachial artery diameter^14^. The response to nitroglycerine was used for assessment of endothelium-independent vasodilation. NID was measured as described previously^14^. Additional details are available in the online-only Data Supplement.

### Statistical analysis

Results are presented as means ± SD for continuous variables and as percentages for categorical variables. Statistical significance was set at a level of P < 0.05. Categorical variables were compared by means of the χ^2^ test. Continuous variables were compared by using one-way analysis of variance (ANOVA) with Tukey’s test for post-hoc comparisons for multiple groups. Associations between variables were determined by Spearman rank correlation analysis^38^. The normal range for WBC count includes ranges for individuals aged 65 years or older and individuals under 65 years of age because WBC count is affected by age^39,40^. We divided the subjects into two groups according to cardiovascular risk factors including age (< 65 years old and ≥ 65 years old), gender, body mass index (BMI) (< 25 kg/m^2^ and ≥ 25 kg/m^2^), hypertension, dyslipidemia, diabetes mellitus and current smoking. In addition, we divided the subjects into three tertiles according to WBC count (≤ 5.2 × 10^3^/μL group, 5.2–6.5 × 10^3^/μL group and > 6.5 × 10^3^/μL group). Since some individuals in the < 65 years of age group will undoubtedly be at the higher end of the age range. Therefore, we additionally divided the subjects into six groups according to age (< 35 years old, 35–44 years old, 45–54 years old, 55–64 years old, 65–74 years old, and ≥ 75 years old). Multiple linear regression analyses were performed to identify independent variables associated with FMD and NID. Adjustment variables for multivariable logistic regression models included age and gender (model 1) and age, gender, BMI, current smoking, presence of hypertension, dyslipidemia, or diabetes mellitus and baseline BAD (model 2). Multiple linear regression analyses were performed to identify independent variables associated with baseline BAD. Adjustment variables for multivariable logistic regression models included age and gender (model 1) and age, gender, BMI, current smoking and presence of hypertension, dyslipidemia or diabetes mellitus (model 2). The data were processed using JMP pro version 13 (SAS institute. Cary, NC).

## Supplementary Information

Supplementary Information.

## Data Availability

All date is available for any reasonable request from the corresponding author.
