# Supplementary material for: White blood cell count is not associated with flow-mediated vasodilation or nitroglycerine-induced vasodilation
Source: Sci Rep. 2022 May 17;12:8201. doi: 10.1038/s41598-022-12205-5 (PMC9114000; doi:10.1038/s41598-022-12205-5)
Supplement: Supplementary file 1 — Supplementary Information. [file 41598_2022_12205_MOESM1_ESM.docx]

**Online Supplement**

**White Blood Cell Count Is Not Associated with Flow-mediated Vasodilation or Nitroglycerine-induced Vasodilation**

Brief title: WBC and vascular function

Shinji Kishimoto, MD, PhD;^1^ Tatsuya Maruhashi, MD, PhD;^1^ Masato Kajikawa, MD, PhD;^2^ Takahiro Harada, MD;^3^ Takayuki Yamaji, MD;^3^ Yiming Han, MS;^1^ Aya Mizobuchi, MS;^1^ Yu Hashimoto, MD;^3^ Kenichi Yoshimura, PhD;^2^ Yukiko Nakano, MD, PhD;^3^ Kazuaki Chayama, MD, PhD;^4^ Chikara Goto, PhD;^5^ Farina Mohamad Yusoff, MD, PhD;^1^ Ayumu Nakashima, MD, PhD;^6^ Yukihito Higashi, MD, PhD, FAHA^1,2^

^1^Department of Cardiovascular Regeneration and Medicine, Research Institute for Radiation Biology and Medicine, Hiroshima University, Hiroshima, Japan

^2^Division of Regeneration and Medicine, Medical Center for Translational and Clinical Research, Hiroshima University Hospital, Hiroshima, Japan

^3^Department of Cardiovascular Medicine, Graduate School of Biomedical and Health Sciences, Hiroshima University, Hiroshima, Japan

^4^Department of Gastroenterology and Metabolism, Graduate School of Biomedical and Health Sciences, Hiroshima University Hiroshima, Japan

^5^Dpartment of Rehabilitation, Faculty of General Rehabilitation, Hiroshima International University, Hiroshima, Japan

^6^Department of Stem Cell Biology and Medicine, Graduate School of Biomedical and Health Sciences, Hiroshima University Hiroshima, Japan

Address for correspondence: Yukihito Higashi, MD, PhD, FAHA

Department of Cardiovascular Regeneration and Medicine,

Research Institute for Radiation Biology and Medicine, Hiroshima University

1-2-3 Kasumi, Minami-ku, Hiroshima 734-8551, Japan

Phone: +81-82-257-5831 Fax: +81-82-257-5831

E-mail: yhigashi@hiroshima-u.ac.jp

**Methods**

**Measurements of FMD and NID**

Vascular response to reactive hyperemia in the brachial artery was used for assessment of endothelium-dependent FMD. A high-resolution linear artery transducer was coupled to computer-assisted analysis software (UNEXEF18G, UNEX Co, Nagoya, Japan) that used an automated edge detection system for measurement of brachial artery diameter.^1^ A blood pressure cuff was placed around the forearm. The brachial artery was scanned longitudinally 5-10 cm above the elbow. When the clearest B-mode image of the anterior and posterior intimal interfaces between the lumen and vessel wall was obtained, the transducer was held at the same point throughout the scan by a special probe holder (UNEX Co) to ensure consistency of the image. Depth and gain setting were set to optimize the images of the arterial lumen wall interface. When the tracking gate was placed on the intima, the artery diameter was automatically tracked, and the waveform of diameter changes over the cardiac cycle was displayed in real time using the FMD mode of the tracking system. This allowed the ultrasound images to be optimized at the start of the scan and the transducer position to be adjusted immediately for optimal tracking performance throughout the scan. Pulsed Doppler flow was assessed at baseline and during peak hyperemic flow, which was confirmed to occur within 15 seconds after cuff deflation. Blood flow velocity was calculated from the color Doppler data and was displayed as a waveform in real time. The baseline longitudinal image of the artery was acquired for 30 seconds, and then the blood pressure cuff was inflated to 50 mm Hg above systolic pressure for 5 minutes. The longitudinal image of the artery was recorded continuously until 5 minutes after cuff deflation. Pulsed Doppler velocity signals were obtained for 20 seconds at baseline and for 10 seconds immediately after cuff deflation. Changes in brachial artery diameter were immediately expressed as percentage change relative to the vessel diameter before cuff inflation. FMD was automatically calculated as the percentage change in peak vessel diameter from the baseline value. Percentage of FMD [(Peak diameter - Baseline diameter)/Baseline diameter] was used for analysis. Blood flow volume was calculated by multiplying the Doppler flow velocity (corrected for the angle) by heart rate and vessel cross-sectional area (-r2). Reactive hyperemia was calculated as the maximum percentage increase in flow after cuff deflation compared with baseline flow.

The response to nitroglycerine was used for assessment of endothelium-independent vasodilation. NID was measured as described previously.^1^ Briefly, after acquiring baseline rest images for 30 seconds, a sublingual tablet (75 μg nitroglycerine) was given, and images of the artery was recorded continuously until the dilation reached a plateau after administration of nitroglycerine. Subjects who had received nitrate treatment and subjects in whom the sublingually administered nitroglycerine tablet was not dissolved during the measurement were excluded from this study. NID was automatically calculated as a percent change in peak vessel diameter from the baseline value. Percentage of NID [(Peak diameter - Baseline diameter)/Baseline diameter] was used for analysis. Inter- and intra-coefficients of variation for the brachial artery diameter were 1.6% and 1.4%, respectively, in our laboratory.

**References**

1. Maruhashi, T, Soga, J, Fujimura, N, et al., Nitroglycerine-induced vasodilation for assessment of vascular function: a comparison with flow-mediated vasodilation, Arteriosclerosis, thrombosis, and vascular biology, 2013;33:1401-1408.

**Supplemental Tables**

**Supplemental Table S1.** Clinical Characteristics of the Subjects

| Variables | < 65 years old  (n = 625) | 65 years old ≤  (n = 726) | P value |
| --- | --- | --- | --- |
| Age, yr | 51±11 | 73±6 | <0.01 |
| Men, n (%) | 394 (63.0) | 418 (57.6) | 0.04 |
| Body mass index, kg/m^2^ | 25.0±4.5 | 23.5±3.6 | <0.01 |
| Systolic blood pressure, mmHg | 134±20 | 133±19 | 0.31 |
| Diastolic blood pressure, mmHg | 82±13 | 76±11 | <0.01 |
| Heart rate, bpm | 71±12 | 70±13 | 0.02 |
| Total cholesterol, mmol/L | 5.20±1.01 | 4.78±0.93 | <0.01 |
| Triglycerides, mmol/L | 1.81±1.22 | 1.39±0.85 | <0.01 |
| HDL cholesterol, mmol/L | 1.53±0.47 | 1.55±0.44 | 0.16 |
| LDL cholesterol, mmol/L | 3.10±0.93 | 2.74±0.83 | <0.01 |
| Glucose, mmol/L | 6.44±2.39 | 6.72±2.39 | 0.05 |
| Hemoglobin A1c, % | 5.7±0.9 | 5.9±0.9 | <0.01 |
| BUN, mmol/L | 5.00±1.61 | 6.07±2.00 | <0.01 |
| Creatinine, umol/L | 88.4±22.1 | 76.0±27.4 | <0.01 |
| eGFR, ml/min/1.73 m^2^ | 79±19 | 64±17 | <0.01 |
| hs-CRP, mg/dL | 0.14±0.26 | 0.18±0.41 | 0.18 |
| Medical history, n (%) |  |  |  |
| Hypertension | 476 (76.2) | 575 (79.2) | 0.18 |
| Dyslipidemia | 360 (57.6) | 493 (67.9) | <0.01 |
| Diabetes mellitus | 149 (23.8) | 295 (40.6) | <0.01 |
| Previous coronary heart disease | 51 (8.2) | 159 (21.9) | <0.01 |
| Previous stroke | 33 (5.3) | 61 (8.4) | 0.02 |
| Current smoker, n (%) | 147 (23.5) | 82 (11.3) | <0.01 |
| Medication, n (%) |  |  |  |
| Antiplatelets | 93 (14.9) | 221 (30.4) | <0.01 |
| Calcium channel blockers | 275 (44.0) | 366 (50.4) | 0.02 |
| ACEIs or ARBs | 185 (29.6) | 326 (44.9) | <0.01 |
| β-blockers | 89 (14.2) | 193 (26.6) | <0.01 |
| Diuretics | 48 (7.7) | 113 (15.6) | <0.01 |
| Statins | 155 (24.8) | 345 (47.5) | <0.01 |
| Medically treated diabetes mellitus |  |  |  |
| Any | 100 (16.0) | 207 (28.5) | <0.01 |
| Insulin dependent | 10 (1.6) | 25 (3.4) | 0.03 |
| White blood cells, × 10^3^/μL | 6.14±1.50 | 5.72±1.35 | <0.01 |
| Neutrophils, × 10^3^/μL | 3.62±1.17 | 3.41±1.03 | <0.01 |
| Lymphocytes, × 10^3^/μL | 1.93±0.60 | 1.74±0.58 | <0.01 |
| Monocytes, × 10^3^/μL | 0.34±0.13 | 0.34±0.12 | 0.78 |
| Eosinophils, × 10^3^/μL | 0.17±0.13 | 0.16±0.12 | 0.17 |
| Basophils, × 10^3^/μL | 0.03±0.02 | 0.03±0.02 | <0.01 |
| Baseline BAD, mm | 4.1±0.7 | 4.1±0.7 | 0.41 |
| FMD, % | 4.2±2.9 | 3.2±2.7 | <0.01 |
| NID, % | 13.2±5.9 | 10.4±5.5 | <0.01 |

HDL indicates high-density lipoprotein; LDL, low-density lipoprotein; BUN, blood urea nitrogen; eGFR, estimated-glomerular filtration rate; hs-CRP, high-sensitive C-reactive protein; ACEIs, angiotensin-converting enzyme inhibitors; ARBs, angiotensin II receptor blockers; BAD, brachial artery diameter; FMD, flow-mediated vasodilation; NID, nitroglycerine-induced vasodilation.

Results are presented as means±SD for continuous variables and percentages for categorical variables.

**Supplemental Table S2.** Clinical Characteristics of the Subjects

| Variables | < 35 years old  (n = 73) | 35-44 years old  (n = 89) | 45-54 years old  (n = 189) | 55-64 years old  (n = 274) | 65-74 years old  (n = 438) | 75 years old ≤  (n = 288) | P value |
| --- | --- | --- | --- | --- | --- | --- | --- |
| Age, yr | 29±4 | 40±3 | 50±3 | 61±3 | 69±3 | 79±4 | <0.01 |
| Men, n (%) | 45 (61.6) | 52 (58.4) | 124 (65.6) | 173 (63.1) | 266 (60.7) | 152 (52.8) | 0.04 |
| Body mass index, kg/m^2^ | 23.7±5.4 | 26.7±6.3 | 25.3±3.7 | 25.0±3.9 | 23.7±3.7 | 23.1±3.5 | <0.01 |
| Systolic blood pressure, mmHg | 126±18 | 131±21 | 136±20 | 136±19 | 132±18 | 133±21 | <0.01 |
| Diastolic blood pressure, mmHg | 75±13 | 81±14 | 85±13 | 85±13 | 82±11 | 73±11 | <0.01 |
| Heart rate, bpm | 72±13 | 72±13 | 73±11 | 70±12 | 70±13 | 69±12 | 0.01 |
| Total cholesterol, mmol/L | 4.99±0.88 | 5.12±1.09 | 5.28±0.93 | 5.22±1.06 | 4.91±0.98 | 4.60±0.78 | <0.01 |
| Triglycerides, mmol/L | 2.12±1.74 | 1.60±0.85 | 1.81±1.31 | 1.81±1.08 | 1.47±0.95 | 1.29±0.64 | <0.01 |
| HDL cholesterol, mmol/L | 1.40±0.39 | 1.45±0.36 | 1.53±0.49 | 1.55±0.49 | 1.55±0.44 | 1.55±0.44 | 0.05 |
| LDL cholesterol, mmol/L | 2.97±0.80 | 3.15±1.01 | 3.18±0.93 | 3.08±0.93 | 2.82±0.85 | 2.61±0.72 | <0.01 |
| Glucose, mmol/L | 5.83±1.89 | 6.00±1.61 | 6.33±1.83 | 6.83±2.94 | 6.66±2.33 | 6.83±2.44 | <0.01 |
| Hemoglobin A1c, % | 5.5±1.1 | 5.5±0.9 | 5.6±1.0 | 5.9±0.8 | 5.9±1.0 | 5.8±0.8 | <0.01 |
| BUN, mmol/L | 4.64±1.43 | 4.64±1.43 | 5.00±1.43 | 5.36±1.79 | 5.71±1.79 | 6.78±2.14 | <0.01 |
| Creatinine, umol/L | 64.5±20.3 | 69.0±28.3 | 69.8±25.6 | 71.6±22.1 | 74.3±28.3 | 79.6±26.5 | <0.01 |
| eGFR, ml/min/1.73 m^2^ | 98±24 | 85±18 | 78±18 | 72±16 | 68±17 | 59±15 | <0.01 |
| hs-CRP, mg/dL | 0.22±0.29 | 0.16±0.48 | 0.12±0.19 | 0.12±0.21 | 0.14±0.32 | 0.26±0.52 | 0.08 |
| Medical history, n (%) |  |  |  |  |  |  |  |
| Hypertension | 39 (53.4) | 66 (74.2) | 154 (81.5) | 217 (79.2) | 344 (78.5) | 231 (80.2) | <0.01 |
| Dyslipidemia | 25 (34.3) | 41 (46.1) | 108 (57.1) | 186 (67.9) | 311 (71.0) | 182 (63.2) | <0.01 |
| Diabetes mellitus | 6 (8.2) | 12 (13.5) | 30 (15.9) | 101 (36.9) | 181 (41.3) | 114 (39.6) | <0.01 |
| Previous coronary heart disease | 0 (0.0) | 3 (3.4) | 11 (5.8) | 37 (13.5) | 98 (22.4) | 61 (21.2) | <0.01 |
| Previous stroke | 1 (1.4) | 6 (6.7) | 6 (3.2) | 20 (7.3) | 32 (7.3) | 29 (10.1) | 0.01 |
| Current smoker, n (%) | 9 (12.3) | 21 (23.6) | 50 (26.5) | 67 (24.5) | 67 (15.3) | 15 (5.2) | <0.01 |
| Medication, n (%) |  |  |  |  |  |  |  |
| Antiplatelets | 6 (8.2) | 10 (11.2) | 20 (10.6) | 57 (20.8) | 121 (27.6) | 100 (34.7) | <0.01 |
| Calcium channel blockers | 21 (28.8) | 45 (50.6) | 91 (48.2) | 118 (43.1) | 213 (48.6) | 153 (53.1) | 0.06 |
| ACEIs or ARBs | 14 (19.2) | 24 (27.0) | 47 (24.9) | 100 (36.5) | 175 (40.0) | 151 (52.4) | <0.01 |
| β-blockers | 6 (8.2) | 14 (15.7) | 16 (8.5) | 53 (19.3) | 114 (26.0) | 79 (274) | <0.01 |
| Diuretics | 2 (2.7) | 5 (5.6) | 10 (5.3) | 31 (11.3) | 53 (12.1) | 60 (20.8) | <0.01 |
| Statins | 1 (1.4) | 12 (13.5) | 29 (15.3) | 113 (41.2) | 205 (26.8) | 140 (48.6) | <0.01 |
| Medically treated diabetes mellitus |  |  |  |  |  |  |  |
| Any | 5 (6.9) | 10 (11.2) | 14 (7.4) | 71 (25.9) | 120 (27.4) | 87 (30.2) | <0.01 |
| Insulin dependent | 0 (0.0) | 1 (1.1) | 2 (1.1) | 7 (2.2) | 15 (3.4) | 10 (3.5) | 0.11 |
| White blood cells, × 10^3^/μL | 5.99±1.80 | 6.33±1.42 | 6.19±1.54 | 6.08±1.40 | 5.76±1.38 | 5.66±1.31 | <0.01 |
| Neutrophils, × 10^3^/μL | 3.57±1.38 | 3.89±1.10 | 3.63±1.19 | 3.55±1.11 | 3.41±1.00 | 3.41±1.06 | <0.01 |
| Lymphocytes, × 10^3^/μL | 1.92±0.57 | 1.79±0.56 | 1.94±0.62 | 1.96±0.60 | 1.78±0.57 | 1.69±0.58 | <0.01 |
| Monocytes, × 10^3^/μL | 0.34±0.14 | 0.36±0.13 | 0.33±0.12 | 0.35±0.13 | 0.34±0.12 | 0.34±0.11 | 0.56 |
| Eosinophils, × 10^3^/μL | 0.19±0.14 | 0.19±0.17 | 0.18±0.12 | 0.16±0.13 | 0.16±0.12 | 0.17±0.13 | 0.37 |
| Basophils, × 10^3^/μL | 0.03±0.02 | 0.03±0.02 | 0.03±0.02 | 0.03±0.02 | 0.03±0.02 | 0.02±0.02 | <0.01 |
| Baseline BAD, mm | 3.8±0.6 | 4.0±0.7 | 4.2±0.7 | 4.1±0.7 | 4.1±0.7 | 4.1±0.7 | <0.01 |
| FMD, % | 5.7±2.5 | 5.2±3.5 | 4.0±2.9 | 3.7±2.6 | 3.5±2.7 | 2.9±2.6 | <0.01 |
| NID, % | 15.1±6.3 | 13.9±5.8 | 12.2±5.1 | 13.2±6.2 | 11.4±5.4 | 8.7±5.4 | <0.01 |

HDL indicates high-density lipoprotein; LDL, low-density lipoprotein; BUN, blood urea nitrogen; eGFR, estimated-glomerular filtration rate; hs-CRP, high-sensitive C-reactive protein; ACEIs, angiotensin-converting enzyme inhibitors; ARBs, angiotensin II receptor blockers; BAD, brachial artery diameter; FMD, flow-mediated vasodilation; NID, nitroglycerine-induced vasodilation.

Results are presented as means±SD for continuous variables and percentages for categorical variables.

**Supplemental Table S3.** Clinical Characteristics of the Subjects

| Variables | Men  (n = 812) | Women  (n = 539) | P value |
| --- | --- | --- | --- |
| Age, yr | 62±14 | 64±14 | 0.02 |
| Men, n (%) | 812 (100.0) | 0 (0.0) | <0.01 |
| Body mass index, kg/m^2^ | 24.7±3.9 | 23.4±4.4 | <0.01 |
| Systolic blood pressure, mmHg | 134±19 | 133±20 | 0.49 |
| Diastolic blood pressure, mmHg | 80±12 | 77±12 | <0.01 |
| Heart rate, bpm | 69±13 | 72±12 | <0.01 |
| Total cholesterol, mmol/L | 4.89±0.96 | 5.15±1.01 | <0.01 |
| Triglycerides, mmol/L | 1.70±1.19 | 1.40±0.77 | <0.01 |
| HDL cholesterol, mmol/L | 1.45±0.44 | 1.66±0.44 | <0.01 |
| LDL cholesterol, mmol/L | 2.84±0.85 | 3.00±0.93 | <0.01 |
| Glucose, mmol/L | 6.77±2.50 | 6.27±2.16 | <0.01 |
| Hemoglobin A1c, % | 5.8±1.0 | 5.7±0.8 | 0.08 |
| BUN, mmol/L | 5.71±1.93 | 5.36±1.82 | <0.01 |
| Creatinine, umol/L | 81.3±25.6 | 61.0±19.5 | <0.01 |
| eGFR, ml/min/1.73 m^2^ | 71±20 | 71±19 | 0.51 |
| hs-CRP, mg/dL | 0.17±0.38 | 0.14±0.27 | 0.30 |
| Medical history, n (%) |  |  |  |
| Hypertension | 631 (77.7) | 420 (77.9) | 0.91 |
| Dyslipidemia | 500 (61.6) | 353 (65.5) | 0.18 |
| Diabetes mellitus | 272 (33.5) | 172 (31.9) | 0.52 |
| Previous coronary heart disease | 171 (21.1) | 39 (7.2) | <0.01 |
| Previous stroke | 70 (5.6) | 24 (4.5) | <0.01 |
| Current smoker, n (%) | 188 (23.2) | 41 (7.6) | <0.01 |
| Medication, n (%) |  |  |  |
| Antiplatelets | 225 (27.7) | 89 (16.5) | <0.01 |
| Calcium channel blockers | 381 (46.9) | 260 (48.2) | 0.71 |
| ACEIs or ARBs | 322 (39.7) | 189 (35.1) | 0.07 |
| β-blockers | 194 (23.9) | 88 (16.3) | <0.01 |
| Diuretics | 107 (13.2) | 54 (10.0) | 0.07 |
| Statins | 288 (35.5) | 212 (39.3) | 0.15 |
| Medically treated diabetes mellitus |  |  |  |
| Any | 178 (21.9) | 129 (23.9) | 0.43 |
| Insulin dependent | 24 (3.0) | 11 (2.0) | 0.28 |
| White blood cells, × 10^3^/μL | 6.11±1.39 | 5.61±1.44 | <0.01 |
| Neutrophils, × 10^3^/μL | 3.62±1.09 | 3.34±1.09 | <0.01 |
| Lymphocytes, × 10^3^/μL | 1.85±0.60 | 1.80±0.59 | 0.24 |
| Monocytes, × 10^3^/μL | 0.37±0.13 | 0.29±0.09 | <0.01 |
| Eosinophils, × 10^3^/μL | 0.19±0.14 | 0.14±0.11 | <0.01 |
| Basophils, × 10^3^/μL | 0.03±0.02 | 0.03±0.02 | <0.01 |
| Baseline BAD, mm | 4.4±0.6 | 3.6±0.6 | <0.01 |
| FMD, % | 3.5±2.6 | 4.0±3.1 | <0.01 |
| NID, % | 11.7±5.8 | 11.9±5.9 | 0.67 |

HDL indicates high-density lipoprotein; LDL, low-density lipoprotein; BUN, blood urea nitrogen; eGFR, estimated-glomerular filtration rate; hs-CRP, high-sensitive C-reactive protein; ACEIs, angiotensin-converting enzyme inhibitors; ARBs, angiotensin II receptor blockers; BAD, brachial artery diameter; FMD, flow-mediated vasodilation; NID, nitroglycerine-induced vasodilation.

Results are presented as means±SD for continuous variables and percentages for categorical variables.

**Supplemental Table S4.** Clinical Characteristics of the Subjects

| Variables | BMI  < 25 kg/m^2^  (n = 840) | BMI  25 kg/m^2^ ≤  (n = 501) | P value |
| --- | --- | --- | --- |
| Age, yr | 64±14 | 60±14 | <0.01 |
| Men, n (%) | 471 (56.1) | 333 (66.5) | <0.01 |
| Body mass index, kg/m^2^ | 21.7±2.3 | 28.3±3.2 | <0.01 |
| Systolic blood pressure, mmHg | 131±20 | 137±18 | <0.01 |
| Diastolic blood pressure, mmHg | 77±12 | 81±12 | <0.01 |
| Heart rate, bpm | 70±12 | 72±12 | <0.01 |
| Total cholesterol, mmol/L | 4.99±0.98 | 4.99±0.93 | 0.98 |
| Triglycerides, mmol/L | 1.39±0.85 | 1.91±1.25 | <0.01 |
| HDL cholesterol, mmol/L | 1.63±0.47 | 1.37±0.36 | <0.01 |
| LDL cholesterol, mmol/L | 2.90±0.91 | 2.95±0.85 | 0.21 |
| Glucose, mmol/L | 6.44±2.22 | 6.83±2.61 | <0.01 |
| Hemoglobin A1c, % | 5.7±0.9 | 5.9±1.0 | <0.01 |
| BUN, mmol/L | 5.71±2.00 | 5.71±1.86 | 0.25 |
| Creatinine, umol/L | 72.5±26.5 | 74.3±23.9 | 0.15 |
| eGFR, ml/min/1.73 m^2^ | 71±20 | 72±19 | 0.48 |
| hs-CRP, mg/dL | 0.16±0.34 | 0.17±0.36 | 0.60 |
| Medical history, n (%) |  |  |  |
| Hypertension | 599 (71.3) | 448 (89.4) | <0.01 |
| Dyslipidemia | 498 (59.3) | 349 (69.7) | <0.01 |
| Diabetes mellitus | 241 (28.7) | 202 (40.3) | <0.01 |
| Previous coronary heart disease | 135 (16.1) | 72 (14.4) | 0.40 |
| Previous stroke | 55 (6.6) | 37 (7.4) | 0.56 |
| Current smoker, n (%) | 140 (16.7) | 89 (17.8) | 0.61 |
| Medication, n (%) |  |  |  |
| Antiplatelets | 204 (24.3) | 106 (21.2) | 0.19 |
| Calcium channel blockers | 339 (40.4) | 301 (60.1) | <0.01 |
| ACEIs or ARBs | 284 (33.8) | 223 (44.5) | <0.01 |
| β-blockers | 176 (21.0) | 102 (20.4) | 0.80 |
| Diuretics | 84 (10.0) | 75 (15.0) | <0.01 |
| Statins | 297 (35.4) | 198 (39.5) | 0.13 |
| Medically treated diabetes mellitus |  |  |  |
| Any | 170 (20.2) | 137 (27.4) | <0.01 |
| Insulin dependent | 25 (3.0) | 10 (2.0) | 0.27 |
| White blood cells, × 10^3^/μL | 5.74±1.42 | 6.20±1.41 | <0.01 |
| Neutrophils, × 10^3^/μL | 3.44±1.10 | 3.61±1.09 | <0.01 |
| Lymphocytes, × 10^3^/μL | 1.75±0.56 | 1.96±0.63 | <0.01 |
| Monocytes, × 10^3^/μL | 0.33±0.12 | 0.35±0.12 | <0.01 |
| Eosinophils, × 10^3^/μL | 0.16±0.13 | 0.18±0.13 | 0.08 |
| Basophils, × 10^3^/μL | 0.03±0.02 | 0.03±0.02 | 0.10 |
| Baseline BAD, mm | 4.0±0.7 | 4.3±0.7 | <0.01 |
| FMD, % | 3.8±2.9 | 3.6±2.8 | 0.25 |
| NID, % | 11.8±6.0 | 11.7±5.7 | 0.87 |

HDL indicates high-density lipoprotein; LDL, low-density lipoprotein; BUN, blood urea nitrogen; eGFR, estimated-glomerular filtration rate; hs-CRP, high-sensitive C-reactive protein; ACEIs, angiotensin-converting enzyme inhibitors; ARBs, angiotensin II receptor blockers; BAD, brachial artery diameter; FMD, flow-mediated vasodilation; NID, nitroglycerine-induced vasodilation.

Results are presented as means±SD for continuous variables and percentages for categorical variables.

**Supplemental Table S5.** Clinical Characteristics of the Subjects

| Variables | Without hypertension  (n = 300) | With hypertension  (n = 1051) | P value |
| --- | --- | --- | --- |
| Age, yr | 60±17 | 64±13 | <0.01 |
| Men, n (%) | 181 (60.3) | 631 (60.0) | 0.93 |
| Body mass index, kg/m^2^ | 22.3±3.6 | 24.7±4.1 | <0.01 |
| Systolic blood pressure, mmHg | 122±16 | 137±19 | <0.01 |
| Diastolic blood pressure, mmHg | 73±11 | 80±12 | <0.01 |
| Heart rate, bpm | 70±13 | 71±12 | 0.17 |
| Total cholesterol, mmol/L | 5.09±1.14 | 4.97±0.93 | 0.05 |
| Triglycerides, mmol/L | 1.43±0.79 | 1.63±1.12 | <0.01 |
| HDL cholesterol, mmol/L | 1.58±0.47 | 1.53±0.44 | 0.05 |
| LDL cholesterol, mmol/L | 2.87±0.83 | 2.87±0.83 | <0.01 |
| Glucose, mmol/L | 6.49±2.28 | 6.61±2.39 | 0.39 |
| Hemoglobin A1c, % | 5.7±0.7 | 5.8±1.0 | 0.06 |
| BUN, mmol/L | 5.71±1.64 | 5.71±1.96 | 0.04 |
| Creatinine, umol/L | 68.1±17.7 | 74.3±27.4 | <0.01 |
| eGFR, ml/min/1.73 m^2^ | 76±20 | 70±19 | <0.01 |
| hs-CRP, mg/dL | 0.13±0.26 | 0.17±0.37 | 0.33 |
| Medical history, n (%) |  |  |  |
| Hypertension | 0 (0.0) | 1051 (100.0) | <0.01 |
| Dyslipidemia | 174 (58.0) | 679 (64.6) | 0.04 |
| Diabetes mellitus | 81 (27.0) | 363 (34.5) | 0.01 |
| Previous coronary heart disease | 42 (14.0) | 200 (19.0) | 0.40 |
| Previous stroke | 13 (4.3) | 81 (7.7) | 0.03 |
| Current smoker, n (%) | 56 (18.7) | 173 (16.5) | 0.37 |
| Medication, n (%) |  |  |  |
| Antiplatelets | 79 (26.3) | 235 (22.3) | 0.15 |
| Calcium channel blockers | 21 (7.0) | 620 (59.0) | <0.01 |
| ACEIs or ARBs | 32 (10.7) | 479 (45.6) | <0.01 |
| β-blockers | 53 (17.7) | 229 (21.8) | <0.01 |
| Diuretics | 25 (8.3) | 136 (12.9) | 0.02 |
| Statins | 98 (32.7) | 402 (38.2) | 0.07 |
| Medically treated diabetes mellitus |  |  |  |
| Any | 48 (16.0) | 259 (24.6) | <0.01 |
| Insulin dependent | 5 (1.7) | 30 (2.9) | 0.23 |
| White blood cells, × 10^3^/μL | 5.80±1.50 | 5.95±1.41 | 0.12 |
| Neutrophils, × 10^3^/μL | 3.42±1.08 | 3.53±1.11 | 0.17 |
| Lymphocytes, × 10^3^/μL | 1.81±0.57 | 1.84±0.60 | 0.64 |
| Monocytes, × 10^3^/μL | 0.34±0.12 | 0.34±0.12 | 0.63 |
| Eosinophils, × 10^3^/μL | 0.18±0.13 | 0.17±0.13 | 0.90 |
| Basophils, × 10^3^/μL | 0.03±0.02 | 0.03±0.02 | 0.93 |
| Baseline BAD, mm | 4.0±0.7 | 4.1±0.7 | <0.01 |
| FMD, % | 4.6±3.0 | 3.5±2.7 | <0.01 |
| NID, % | 13.7±6.2 | 11.2±5.6 | <0.01 |

HDL indicates high-density lipoprotein; LDL, low-density lipoprotein; BUN, blood urea nitrogen; eGFR, estimated-glomerular filtration rate; hs-CRP, high-sensitive C-reactive protein; ACEIs, angiotensin-converting enzyme inhibitors; ARBs, angiotensin II receptor blockers; BAD, brachial artery diameter; FMD, flow-mediated vasodilation; NID, nitroglycerine-induced vasodilation.

Results are presented as means±SD for continuous variables and percentages for categorical variables.

**Supplemental Table S6.** Clinical Characteristics of the Subjects

| Variables | Without dyslipidemia  (n = 498) | With dyslipidemia  (n = 853) | P value |
| --- | --- | --- | --- |
| Age, yr | 60±17 | 64±12 | <0.01 |
| Men, n (%) | 312 (62.7) | 500 (58.6) | 0.14 |
| Body mass index, kg/m^2^ | 23.3±3.7 | 24.7±4.3 | <0.01 |
| Systolic blood pressure, mmHg | 132±19 | 134±20 | 0.05 |
| Diastolic blood pressure, mmHg | 79±12 | 79±12 | 0.56 |
| Heart rate, bpm | 70±12 | 71±12 | 0.60 |
| Total cholesterol, mmol/L | 4.81±0.70 | 5.07±1.09 | <0.01 |
| Triglycerides, mmol/L | 1.32±0.85 | 1.74±1.13 | <0.01 |
| HDL cholesterol, mmol/L | 1.63±0.47 | 1.47±0.44 | <0.01 |
| LDL cholesterol, mmol/L | 2.77±0.65 | 3.00±0.98 | <0.01 |
| Glucose, mmol/L | 6.16±1.89 | 6.83±2.61 | <0.01 |
| Hemoglobin A1c, % | 5.5±0.7 | 5.9±1.0 | <0.01 |
| BUN, mmol/L | 5.36±1.79 | 5.71±1.96 | <0.01 |
| Creatinine, umol/L | 71.6±27.4 | 74.3±23.9 | 0.16 |
| eGFR, ml/min/1.73 m^2^ | 75±21 | 69±18 | <0.01 |
| hs-CRP, mg/dL | 0.13±0.27 | 0.18±0.38 | 0.10 |
| Medical history, n (%) |  |  |  |
| Hypertension | 372 (74.7) | 679 (79.6) | 0.04 |
| Dyslipidemia | 0 (0.0) | 853 (100.0) | <0.01 |
| Diabetes mellitus | 84 (16.9) | 360 (42.2) | <0.01 |
| Previous coronary heart disease | 21 (4.2) | 189 (22.2) | <0.01 |
| Previous stroke | 30 (6.0) | 64 (7.5) | 0.30 |
| Current smoker, n (%) | 96 (19.3) | 133 (15.6) | 0.08 |
| Medication, n (%) |  |  |  |
| Antiplatelets | 61 (12.3) | 253 (29.7) | <0.01 |
| Calcium channel blockers | 217 (43.6) | 424 (49.7) | 0.03 |
| ACEIs or ARBs | 160 (32.1) | 351 (41.1) | <0.01 |
| β-blockers | 68 (13.7) | 214 (25.1) | <0.01 |
| Diuretics | 41 (8.2) | 120 (14.1) | <0.01 |
| Statins | 17 (3.4) | 483 (56.6) | <0.01 |
| Medically treated diabetes mellitus |  |  |  |
| Any | 50 (10.0) | 257 (30.1) | <0.01 |
| Insulin dependent | 7 (1.4) | 28 (3.3) | 0.03 |
| White blood cells, × 10^3^/μL | 5.79±1.37 | 5.99±1.46 | 0.02 |
| Neutrophils, × 10^3^/μL | 3.43±1.07 | 3.56±1.12 | 0.05 |
| Lymphocytes, × 10^3^/μL | 1.78±0.56 | 1.86±0.62 | 0.03 |
| Monocytes, × 10^3^/μL | 0.33±0.12 | 0.34±0.12 | 0.12 |
| Eosinophils, × 10^3^/μL | 0.17±0.13 | 0.17±0.13 | 0.98 |
| Basophils, × 10^3^/μL | 0.03±0.02 | 0.03±0.02 | 0.89 |
| Baseline BAD, mm | 4.1±0.7 | 4.1±0.7 | 0.27 |
| FMD, % | 3.8±2.9 | 3.7±2.7 | 0.37 |
| NID, % | 12.0±5.6 | 11.6±6.0 | 0.30 |

HDL indicates high-density lipoprotein; LDL, low-density lipoprotein; BUN, blood urea nitrogen; eGFR, estimated-glomerular filtration rate; hs-CRP, high-sensitive C-reactive protein; ACEIs, angiotensin-converting enzyme inhibitors; ARBs, angiotensin II receptor blockers; BAD, brachial artery diameter; FMD, flow-mediated vasodilation; NID, nitroglycerine-induced vasodilation.

Results are presented as means±SD for continuous variables and percentages for categorical variables.

**Supplemental Table S7.** Clinical Characteristics of the Subjects

| Variables | Without diabetes mellitus  (n = 907) | With diabetes mellitus  (n = 444) | P value |
| --- | --- | --- | --- |
| Age, yr | 61±15 | 67±10 | <0.01 |
| Men, n (%) | 540 (59.5) | 272 (61.3) | 0.54 |
| Body mass index, kg/m^2^ | 23.7±3.8 | 25.1±4.6 | <0.01 |
| Systolic blood pressure, mmHg | 133±20 | 134±19 | 0.33 |
| Diastolic blood pressure, mmHg | 80±13 | 77±11 | <0.01 |
| Heart rate, bpm | 70±13 | 71±12 | 0.17 |
| Total cholesterol, mmol/L | 5.07±1.01 | 4.78±0.93 | <0.01 |
| Triglycerides, mmol/L | 1.52±0.98 | 1.69±1.16 | <0.01 |
| HDL cholesterol, mmol/L | 1.58±0.47 | 1.47±0.44 | <0.01 |
| LDL cholesterol, mmol/L | 3.00±0.93 | 2.72±0.80 | <0.01 |
| Glucose, mmol/L | 5.88±1.22 | 8.05±3.33 | <0.01 |
| Hemoglobin A1c, % | 5.4±0.4 | 6.5±1.1 | <0.01 |
| BUN, mmol/L | 5.36±1.61 | 6.07±2.32 | <0.01 |
| Creatinine, umol/L | 70.7±23.9 | 77.8±30.9 | <0.01 |
| eGFR, ml/min/1.73 m^2^ | 73±19 | 67±20 | <0.01 |
| hs-CRP, mg/dL | 0.16±0.36 | 0.18±0.27 | 0.58 |
| Medical history, n (%) |  |  |  |
| Hypertension | 688 (75.9) | 363 (81.8) | 0.01 |
| Dyslipidemia | 493 (54.4) | 360 (81.1) | <0.01 |
| Diabetes mellitus | 0 (0.0) | 444 (100.0) | <0.01 |
| Previous coronary heart disease | 105 (11.6) | 105 (23.6) | <0.01 |
| Previous stroke | 58 (6.4) | 36 (8.1) | 0.25 |
| Current smoker, n (%) | 141 (15.6) | 88 (19.8) | 0.05 |
| Medication, n (%) |  |  |  |
| Antiplatelets | 167 (18.4) | 147 (33.1) | <0.01 |
| Calcium channel blockers | 386 (42.6) | 255 (57.4) | <0.01 |
| ACEIs or ARBs | 295 (32.5) | 216 (48.6) | <0.01 |
| β-blockers | 170 (18.7) | 112 (25.2) | <0.01 |
| Diuretics | 85 (9.4) | 76 (17.1) | <0.01 |
| Statins | 241 (26.6) | 259 (58.3) | <0.01 |
| Medically treated diabetes mellitus |  |  |  |
| Any | 0 (0.0) | 307 (69.1) | <0.01 |
| Insulin dependent | 0 (0.0) | 35 (7.9) | 0.03 |
| White blood cells, × 10^3^/μL | 5.81±1.40 | 6.13±1.48 | <0.01 |
| Neutrophils, × 10^3^/μL | 3.42±1.08 | 3.71±1.14 | <0.01 |
| Lymphocytes, × 10^3^/μL | 1.81±0.59 | 1.89±0.60 | 0.03 |
| Monocytes, × 10^3^/μL | 0.33±0.11 | 0.36±0.14 | <0.01 |
| Eosinophils, × 10^3^/μL | 0.16±0.12 | 0.18±0.15 | 0.03 |
| Basophils, × 10^3^/μL | 0.03±0.02 | 0.03±0.02 | 0.42 |
| Baseline BAD, mm | 4.1±0.7 | 4.2±0.6 | 0.04 |
| FMD, % | 4.0±2.9 | 3.2±2.6 | <0.01 |
| NID, % | 12.2±5.9 | 10.8±5.7 | <0.01 |

HDL indicates high-density lipoprotein; LDL, low-density lipoprotein; BUN, blood urea nitrogen; eGFR, estimated-glomerular filtration rate; hs-CRP, high-sensitive C-reactive protein; ACEIs, angiotensin-converting enzyme inhibitors; ARBs, angiotensin II receptor blockers; BAD, brachial artery diameter; FMD, flow-mediated vasodilation; NID, nitroglycerine-induced vasodilation.

Results are presented as means±SD for continuous variables and percentages for categorical variables.

**Supplemental Table S8.** Clinical Characteristics of the Subjects

| Variables | Non-smoker  (n = 1122) | Current smoker  (n = 229) | P value |
| --- | --- | --- | --- |
| Age, yr | 64±14 | 58±12 | <0.01 |
| Men, n (%) | 624 (55.6) | 188 (82.1) | 0.54 |
| Body mass index, kg/m^2^ | 24.1±4.1 | 24.7±4.5 | 0.06 |
| Systolic blood pressure, mmHg | 133±19 | 136±22 | 0.02 |
| Diastolic blood pressure, mmHg | 78±12 | 81±13 | <0.01 |
| Heart rate, bpm | 70±12 | 71±13 | 0.57 |
| Total cholesterol, mmol/L | 4.99±0.98 | 4.99±1.01 | 0.76 |
| Triglycerides, mmol/L | 1.54±0.97 | 1.85±1.35 | <0.01 |
| HDL cholesterol, mmol/L | 1.55±0.44 | 1.47±0.47 | 0.01 |
| LDL cholesterol, mmol/L | 2.92±0.88 | 2.90±0.91 | 0.67 |
| Glucose, mmol/L | 6.55±2.33 | 6.72±2.61 | 0.48 |
| Hemoglobin A1c, % | 5.8±0.9 | 5.9±1.0 | 0.28 |
| BUN, mmol/L | 5.71±1.89 | 5.00±1.75 | <0.01 |
| Creatinine, umol/L | 73.4±25.6 | 72.5±24.8 | 0.74 |
| eGFR, ml/min/1.73 m^2^ | 70±19 | 78±20 | <0.01 |
| hs-CRP, mg/dL | 0.17±0.37 | 0.11±0.19 | 0.19 |
| Medical history, n (%) |  |  |  |
| Hypertension | 878 (78.3) | 173 (75.5) | 0.37 |
| Dyslipidemia | 720 (64.2) | 133 (58.1) | 0.08 |
| Diabetes mellitus | 356 (31.7) | 88 (38.4) | 0.05 |
| Previous coronary heart disease | 184 (16.4) | 26 (11.4) | 0.04 |
| Previous stroke | 84 (7.5) | 10 (4.4) | 0.07 |
| Current smoker, n (%) | 0 (0.0) | 229 (100.0) | <0.01 |
| Medication, n (%) |  |  |  |
| Antiplatelets | 265 (23.6) | 49 (21.3) | 0.46 |
| Calcium channel blockers | 538 (48.0) | 103 (45.0) | 0.41 |
| ACEIs or ARBs | 425 (37.9) | 86 (37.6) | 0.93 |
| β-blockers | 238 (21.2) | 44 (19.2) | 0.49 |
| Diuretics | 140 (12.5) | 21 (9.2) | 0.15 |
| Statins | 428 (38.2) | 72 (31.4) | 0.05 |
| Medically treated diabetes mellitus |  |  |  |
| Any | 245 (21.8) | 62 (27.1) | 0.09 |
| Insulin dependent | 32 (2.9) | 3 (1.3) | 0.15 |
| White blood cells, × 10^3^/μL | 5.77±1.36 | 6.63±1.55 | <0.01 |
| Neutrophils, × 10^3^/μL | 3.44±1.05 | 3.86±1.27 | <0.01 |
| Lymphocytes, × 10^3^/μL | 1.78±0.57 | 2.10±0.64 | <0.01 |
| Monocytes, × 10^3^/μL | 0.33±0.12 | 0.38±0.12 | <0.01 |
| Eosinophils, × 10^3^/μL | 0.16±0.13 | 0.19±0.14 | 0.03 |
| Basophils, × 10^3^/μL | 0.03±0.02 | 0.03±0.02 | <0.01 |
| Baseline BAD, mm | 4.1±0.7 | 4.1±0.7 | 0.59 |
| FMD, % | 3.7±2.8 | 3.8±2.9 | 0.45 |
| NID, % | 11.6±6.0 | 12.4±5.3 | 0.10 |

HDL indicates high-density lipoprotein; LDL, low-density lipoprotein; BUN, blood urea nitrogen; eGFR, estimated-glomerular filtration rate; hs-CRP, high-sensitive C-reactive protein; ACEIs, angiotensin-converting enzyme inhibitors; ARBs, angiotensin II receptor blockers; BAD, brachial artery diameter; FMD, flow-mediated vasodilation; NID, nitroglycerine-induced vasodilation.

Results are presented as means±SD for continuous variables and percentages for categorical variables.

**Supplemental Table S9.** Clinical Characteristics of the Subjects According to White Blood Cell Count

| Variables | Low WBC  ≤ 5.2 × 10^3^/μL  (n = 458) | Middle WBC  5.2-6.5 × 10^3^/μL  (n = 455) | High WBC  6.5 × 10^3^/μL <  (n = 438) | P value |
| --- | --- | --- | --- | --- |
| Age, yr | 64±14 | 64±13 | 60±15 | <0.01 |
| Men, n (%) | 230 (50.2) | 288 (63.3) | 294 (67.1) | <0.01 |
| Body mass index, kg/m^2^ | 23.3±3.9 | 24.3±4.0 | 25.0±4.4 | <0.01 |
| Systolic blood pressure, mmHg | 132±18 | 134±20 | 135±20 | 0.10 |
| Diastolic blood pressure, mmHg | 77±12 | 79±12 | 80±13 | <0.01 |
| Heart rate, bpm | 70±13 | 70±12 | 72±12 | 0.08 |
| Total cholesterol, mmol/L | 4.91±0.93 | 4.94±0.98 | 5.12±1.03 | <0.01 |
| Triglycerides, mmol/L | 1.34±0.84 | 1.60±1.08 | 1.82±1.15 | <0.01 |
| HDL cholesterol, mmol/L | 1.66±0.49 | 1.50±0.44 | 1.45±0.39 | <0.01 |
| LDL cholesterol, mmol/L | 2.82±0.83 | 2.87±0.91 | 3.05±0.93 | <0.01 |
| Glucose, mmol/L | 6.44±2.39 | 6.66±2.33 | 6.66±2.44 | 0.34 |
| Hemoglobin A1c, % | 5.6±0.7 | 5.8±0.9 | 5.9±1.1 | <0.01 |
| BUN, mmol/L | 5.71±1.89 | 5.00±1.75 | 5.00±1.75 | 0.96 |
| Creatinine, umol/L | 69.8±24.8 | 73.4±26.5 | 76.0±28.3 | <0.01 |
| eGFR, ml/min/1.73 m^2^ | 71±18 | 71±19 | 71±21 | 0.90 |
| hs-CRP, mg/dL | 0.10±0.24 | 0.13±0.26 | 0.25±0.46 | <0.01 |
| Medical history, n (%) |  |  |  |  |
| Hypertension | 346 (75.6) | 360 (79.1) | 345 (78.8) | 0.36 |
| Dyslipidemia | 275 (60.0) | 289 (63.5) | 289 (66.0) | 0.18 |
| Diabetes mellitus | 129 (28.2) | 142 (31.2) | 173 (39.5) | <0.01 |
| Previous coronary heart disease | 69 (15.1) | 81 (17.8) | 60 (13.7) | 0.23 |
| Previous stroke | 37 (8.1) | 28 (6.2) | 29 (6.6) | 0.50 |
| Current smoker, n (%) | 47 (10.3) | 61 (13.4) | 121 (27.6) | <0.01 |
| Medication, n (%) |  |  |  |  |
| Antiplatelets | 110 (24.0) | 107 (23.5) | 97 (22.2) | 0.79 |
| Calcium channel blockers | 204 (44.5) | 221 (48.6) | 216 (49.3) | 0.30 |
| ACEIs or ARBs | 174 (38.0) | 164 (36.0) | 173 (39.5) | 0.57 |
| β-blockers | 97 (21.2) | 89 (19.6) | 96 (21.9) | 0.67 |
| Diuretics | 54 (11.8) | 52 (11.4) | 55 (12.6) | 0.87 |
| Statins | 170 (37.1) | 179 (39.3) | 151 (34.5) | 0.32 |
| Medically treated diabetes mellitus |  |  |  |  |
| Any | 92 (20.1) | 100 (22.0) | 115 (26.3) | 0.08 |
| Insulin dependent | 12 (2.6) | 9 (2.0) | 14 (3.2) | 0.51 |
| White blood cells, × 10^3^/μL | 4.41±0.60 | 5.85±0.38 | 7.55±0.89 | <0.01 |
| Neutrophils, × 10^3^/μL | 2.56±0.54 | 3.43±0.54 | 4.64±0.98 | <0.01 |
| Lymphocytes, × 10^3^/μL | 1.45±0.41 | 1.88±0.50 | 2.20±0.61 | <0.01 |
| Monocytes, × 10^3^/μL | 0.27±0.08 | 0.34±0.10 | 0.42±0.13 | <0.01 |
| Eosinophils, × 10^3^/μL | 0.13±0.09 | 0.17±0.12 | 0.21±0.16 | <0.01 |
| Basophils, × 10^3^/μL | 0.02±0.01 | 0.03±0.02 | 0.03±0.02 | <0.01 |
| Baseline BAD, mm | 4.0±0.7 | 4.1±0.7 | 4.1±0.7 | 0.03 |
| FMD, % | 3.9±2.9 | 3.5±2.7 | 3.8±2.8 | 0.06 |
| NID, % | 11.6±5.8 | 11.8±5.9 | 11.9±5.8 | 0.84 |

WBC indicates white blood cell; HDL, high-density lipoprotein; LDL, low-density lipoprotein; BUN, blood urea nitrogen; eGFR, estimated-glomerular filtration rate; hs-CRP, high-sensitive C-reactive protein; ACEIs, angiotensin-converting enzyme inhibitors; ARBs, angiotensin II receptor blockers; BAD, brachial artery diameter; FMD, flow-mediated vasodilation; NID, nitroglycerine-induced vasodilation.

Results are presented as means±SD for continuous variables and percentages for categorical variables.

**Supplemental Table S10.** Clinical Characteristics of the Subjects

| Variables | Low baseline BAD  ≤ 4.09 mm  (n = 681) | High baseline BAD  4.09 mm <  (n = 670) | P value |
| --- | --- | --- | --- |
| Age, yr | 62±15 | 64±13 | 0.08 |
| Men, n (%) | 268 (39.4) | 544 (81.2) | <0.01 |
| Body mass index, kg/m^2^ | 23.3±4.3 | 25.0±3.9 | <0.01 |
| Systolic blood pressure, mmHg | 133±20 | 134±19 | 0.23 |
| Diastolic blood pressure, mmHg | 78±12 | 80±12 | <0.01 |
| Heart rate, bpm | 71±12 | 70±12 | 0.52 |
| Total cholesterol, mmol/L | 5.07±1.03 | 4.89±0.91 | <0.01 |
| Triglycerides, mmol/L | 1.50±0.97 | 1.68±1.13 | <0.01 |
| HDL cholesterol, mmol/L | 1.60±0.47 | 1.47±0.41 | <0.01 |
| LDL cholesterol, mmol/L | 2.95±0.93 | 2.87±0.83 | 0.21 |
| Glucose, mmol/L | 6.38±2.44 | 6.77±2.33 | <0.01 |
| Hemoglobin A1c, % | 5.7±0.9 | 5.9±0.9 | 0.06 |
| BUN, mmol/L | 5.50±1.89 | 5.85±1.93 | <0.01 |
| Creatinine, umol/L | 68.1±23.9 | 78.7±25.6 | <0.01 |
| eGFR, ml/min/1.73 m^2^ | 73±20 | 69±18 | <0.01 |
| hs-CRP, mg/dL | 0.16±0.34 | 0.16±0.35 | 0.91 |
| Medical history, n (%) |  |  |  |
| Hypertension | 502 (73.7) | 549 (81.9) | <0.01 |
| Dyslipidemia | 425 (62.4) | 428 (63.9) | 0.57 |
| Diabetes mellitus | 194 (23.5) | 250 (37.3) | <0.01 |
| Previous coronary heart disease | 83 (12.2) | 127 (19.0) | <0.01 |
| Previous stroke | 50 (7.3) | 44 (6.6) | 0.58 |
| Current smoker, n (%) | 110 (16.2) | 119 (17.8) | 0.43 |
| Medication, n (%) |  |  |  |
| Antiplatelets | 151 (22.2) | 163 (24.3) | 0.35 |
| Calcium channel blockers | 289 (42.4) | 352 (52.5) | <0.01 |
| ACEIs or ARBs | 249 (36.6) | 262 (39.1) | 0.34 |
| β-blockers | 137 (20.1) | 145 (21.6) | 0.49 |
| Diuretics | 75 (11.0) | 86 (12.8) | 0.30 |
| Statins | 250 (26.7) | 250 (37.3) | 0.82 |
| Medically treated diabetes mellitus |  |  |  |
| Any | 139 (20.4) | 168 (25.1) | 0.04 |
| Insulin dependent | 20 (2.9) | 15 (2.2) | 0.42 |
| White blood cells, × 10^3^/μL | 5.79±1.44 | 6.04±1.42 | <0.01 |
| Neutrophils, × 10^3^/μL | 3.47±1.06 | 3.55±1.15 | 0.27 |
| Lymphocytes, × 10^3^/μL | 1.80±0.58 | 1.86±0.61 | 0.12 |
| Monocytes, × 10^3^/μL | 0.32±0.12 | 0.36±0.12 | <0.01 |
| Eosinophils, × 10^3^/μL | 0.16±0.13 | 0.17±0.13 | 0.09 |
| Basophils, × 10^3^/μL | 0.03±0.02 | 0.03±0.02 | 0.05 |
| Baseline BAD, mm | 3.6±0.4 | 4.7±0.4 | <0.01 |
| FMD, % | 4.6±3.0 | 2.8±2.3 | <0.01 |
| NID, % | 13.7±5.9 | 9.8±5.2 | <0.01 |

BAD indicates brachial artery diameter; HDL, high-density lipoprotein; LDL, low-density lipoprotein; BUN, blood urea nitrogen; eGFR, estimated-glomerular filtration rate; hs-CRP, high-sensitive C-reactive protein; ACEIs, angiotensin-converting enzyme inhibitors; ARBs, angiotensin II receptor blockers; FMD, flow-mediated vasodilation; NID, nitroglycerine-induced vasodilation.

Results are presented as means±SD for continuous variables and percentages for categorical variables.

**Supplemental Table S11.** Univariate Analysis of Relationships among White Blood Cell, FMD, NID and Variables in Subjects under 65 Years of Age

| Variables | White blood cell | FMD | NID |
| --- | --- | --- | --- |
| Age, yr | -0.02 | -0.25† | -0.10* |
| Body mass index, kg/m^2^ | 0.29† | -0.06 | -0.02 |
| Systolic blood pressure, mmHg | 0.08* | -0.18† | -0.13† |
| Diastolic blood pressure, mmHg | 0.10† | -0.15† | -0.05 |
| Heart rate, bpm | 0.15† | 0.01 | -0.02 |
| Total cholesterol, mmol/L | 0.11* | -0.04 | 0.08 |
| Triglycerides, mmol/L | 0.18† | -0.12† | 0.01 |
| HDL cholesterol, mmol/L | -0.22† | -0.01 | 0.02 |
| LDL cholesterol, mmol/L | 0.13† | -0.02 | 0.02 |
| Glucose, mmol/L | 0.06 | -0.17† | 0.02 |
| Hemoglobin A1c, % | 0.19† | -0.16† | -0.03 |
| BUN, mmol/L | 0.07 | -0.01 | -0.01 |
| Creatinine, umol/L | 0.16† | -0.05 | -0.04 |
| eGFR, ml/min/1.73 m^2^ | -0.04 | 0.10* | 0.05 |
| hs-CRP, mg/dL | 0.12 | 0.04 | -0.12 |
| Smoking, pack-years | 0.19† | -0.08 | -0.08 |
| White blood cells, × 10^3^/μL | - | -0.06 | -0.01 |
| Neutrophils, × 10^3^/μL | 0.90† | -0.01 | -0.02 |
| Lymphocytes, × 10^3^/μL | 0.56† | -0.10* | -0.01 |
| Monocytes, × 10^3^/μL | 0.61† | -0.04 | -0.02 |
| Eosinophils, × 10^3^/μL | 0.28† | 0.10* | 0.07 |
| Basophils, × 10^3^/μL | 0.22† | -0.05 | -0.08 |
| Baseline BAD, mm | 0.12† | -0.40† | -0.40† |
| FMD, % | -0.06 | - | 0.38† |
| NID, % | -0.01 | 0.38† | - |

*P<0.05, †P<0.01

FMD indicates flow-mediated vasodilation; NID, nitroglycerine-induced vasodilation HDL, high-density lipoprotein; LDL, low-density lipoprotein; BUN, blood urea nitrogen; eGFR, estimated-glomerular filtration rate; hs-CRP, high-sensitive C-reactive protein; BAD, brachial artery diameter.

**Supplemental Table S12.** Univariate Analysis of Relationships among White Blood Cell, FMD, NID and Variables in Subjects with 65 Years of Age and Older

| Variables | White blood cell | FMD | NID |
| --- | --- | --- | --- |
| Age, yr | -0.04 | -0.15† | -0.28† |
| Body mass index, kg/m^2^ | 0.08* | -0.01 | -0.09* |
| Systolic blood pressure, mmHg | 0.03 | -0.04 | -0.09* |
| Diastolic blood pressure, mmHg | 0.01 | 0.03 | 0.06 |
| Heart rate, bpm | -0.01 | 0.02 | -0.01 |
| Total cholesterol, mmol/L | -0.01 | -0.07 | 0.04 |
| Triglycerides, mmol/L | 0.16† | -0.08* | 0.01 |
| HDL cholesterol, mmol/L | -0.20† | 0.01 | 0.02 |
| LDL cholesterol, mmol/L | 0.02 | -0.07 | 0.03 |
| Glucose, mmol/L | 0.05 | -0.10* | -0.07 |
| Hemoglobin A1c, % | 0.11* | -0.07 | -0.09* |
| BUN, mmol/L | 0.04 | -0.05 | -0.13† |
| Creatinine, umol/L | 0.15† | -0.08* | -0.12† |
| eGFR, ml/min/1.73 m^2^ | -0.05 | 0.06 | 0.11† |
| hs-CRP, mg/dL | 0.21† | 0.09 | 0.06 |
| Smoking, pack-years | 0.13* | -0.01 | -0.02 |
| White blood cells, × 10^3^/μL | - | 0.01 | -0.01 |
| Neutrophils, × 10^3^/μL | 0.85† | 0.02 | -0.03 |
| Lymphocytes, × 10^3^/μL | 0.50† | 0.08 | 0.10* |
| Monocytes, × 10^3^/μL | 0.57† | 0.08 | -0.08 |
| Eosinophils, × 10^3^/μL | 0.27† | -0.03 | 0.02 |
| Basophils, × 10^3^/μL | 0.25† | 0.10* | -0.04 |
| Baseline BAD, mm | 0.03 | -0.28† | -0.40† |
| FMD, % | 0.01 | - | 0.38 |
| NID, % | -0.01 | 0.38 | - |

*P<0.05, †P<0.01

FMD indicates flow-mediated vasodilation; NID, nitroglycerine-induced vasodilation HDL, high-density lipoprotein; LDL, low-density lipoprotein; BUN, blood urea nitrogen; eGFR, estimated-glomerular filtration rate; hs-CRP, high-sensitive C-reactive protein; BAD, brachial artery diameter.

**Supplemental Table S13.** Univariate Analysis of Relationships among White Blood Cell, FMD, NID and Variables in Subjects under 35 Years of Age

| Variables | White blood cell | FMD | NID |
| --- | --- | --- | --- |
| Age, yr | -0.01 | -0.21 | -0.09 |
| Body mass index, kg/m^2^ | 0.44† | -0.16 | 0.12 |
| Systolic blood pressure, mmHg | 0.29* | -0.20 | 0.11 |
| Diastolic blood pressure, mmHg | 0.07 | -0.14 | 0.11 |
| Heart rate, bpm | -0.03 | 0.11 | -0.18 |
| Total cholesterol, mmol/L | 0.04 | -0.24 | -0.06 |
| Triglycerides, mmol/L | 0.36† | -.06 | 0.01 |
| HDL cholesterol, mmol/L | -023 | 0.21 | -0.22 |
| LDL cholesterol, mmol/L | 0.02 | -0.25 | -0.04 |
| Glucose, mmol/L | 0.16 | -0.29* | -0.002 |
| Hemoglobin A1c, % | 0.28 | 0.04 | 0.16 |
| BUN, mmol/L | 0.15 | 0.06 | -0.06 |
| Creatinine, umol/L | 0.14 | -0.09 | 0.14 |
| eGFR, ml/min/1.73 m^2^ | 0.04 | -0.03 | -0.15 |
| hs-CRP, mg/dL | -0.04 | -0.32 | -0.39* |
| Smoking, pack-years | 0.07 | -0.13 | -0.21 |
| White blood cells, × 10^3^/μL | - | -0.14 | 0.06 |
| Neutrophils, × 10^3^/μL | 0.90† | 0.04 | -0.04 |
| Lymphocytes, × 10^3^/μL | 0.48† | -0.07 | -0.18 |
| Monocytes, × 10^3^/μL | 0.72† | -0.13 | -0.18 |
| Eosinophils, × 10^3^/μL | 0.29* | -0.14 | -0.11 |
| Basophils, × 10^3^/μL | 0.05 | -0.20 | -0.38† |
| Baseline BAD, mm | 0.15 | -0.31† | -0.26* |
| FMD, % | -0.14 | - | 0.08 |
| NID, % | 0.06 | 0.08 | - |

*P<0.05, †P<0.01

FMD indicates flow-mediated vasodilation; NID, nitroglycerine-induced vasodilation; BAD, brachial artery diameter; HDL, high-density lipoprotein; LDL, low-density lipoprotein; BUN, blood urea nitrogen; eGFR, estimated-glomerular filtration rate; hs-CRP, high-sensitive C-reactive protein.

**Supplemental Table S14.** Univariate Analysis of Relationships among White Blood Cell, FMD, NID and Variables in Subjects with 35-44 Years of Age

| Variables | White blood cell | FMD | NID |
| --- | --- | --- | --- |
| Age, yr | -0.06 | -0.21 | -0.11 |
| Body mass index, kg/m^2^ | 0.30† | 0.10 | 0.11 |
| Systolic blood pressure, mmHg | 0.14 | 0.06 | -010 |
| Diastolic blood pressure, mmHg | 0.19 | 0.03 | -0003 |
| Heart rate, bpm | 0.13 | 0.03 | 0.22* |
| Total cholesterol, mmol/L | 0.17 | 0.07 | 0.24* |
| Triglycerides, mmol/L | 0.20 | -0.14 | 0.13 |
| HDL cholesterol, mmol/L | -0.09 | 0.19 | 0.15 |
| LDL cholesterol, mmol/L | 0.24* | -0.07 | 0.08 |
| Glucose, mmol/L | 0.30† | 0.10 | 0.23 |
| Hemoglobin A1c, % | 0.31† | 0.06 | 0.28* |
| BUN, mmol/L | 0.03 | 0.15 | 0.18 |
| Creatinine, umol/L | 0.16 | -0.11 | -0.01 |
| eGFR, ml/min/1.73 m^2^ | -0.03 | 0.02 | 0.04 |
| hs-CRP, mg/dL | 0.27 | -0.15 | -0.10 |
| Smoking, pack-years | 0.37* | -0.17 | 0.04 |
| White blood cells, × 10^3^/μL | - | -0.17 | -0.09 |
| Neutrophils, × 10^3^/μL | 0.89† | -0.10 | -0.10 |
| Lymphocytes, × 10^3^/μL | 0.59† | -0.05 | 0.23 |
| Monocytes, × 10^3^/μL | 0.58† | -0.11 | 0.04 |
| Eosinophils, × 10^3^/μL | 0.34† | 0.05 | 0.15 |
| Basophils, × 10^3^/μL | 0.15 | -0.23 | -0.11 |
| Baseline BAD, mm | 0.15 | -0.46† | -0.35† |
| FMD, % | -0.17 | - | 0.55† |
| NID, % | -0.09 | 0.55† | - |

*P<0.05, †P<0.01

FMD indicates flow-mediated vasodilation; NID, nitroglycerine-induced vasodilation; BAD, brachial artery diameter; HDL, high-density lipoprotein; LDL, low-density lipoprotein; BUN, blood urea nitrogen; eGFR, estimated-glomerular filtration rate; hs-CRP, high-sensitive C-reactive protein.

**Supplemental Table S15.** Univariate Analysis of Relationships among White Blood Cell, FMD, NID and Variables in Subjects with 45-54 Years of Age

| Variables | White blood cell | FMD | NID |
| --- | --- | --- | --- |
| Age, yr | -0.17* | -0.004 | -0.06 |
| Body mass index, kg/m^2^ | 0.26† | -0.12 | -0.05 |
| Systolic blood pressure, mmHg | 0.12 | -0.22† | -0.18* |
| Diastolic blood pressure, mmHg | 0.15* | -0.17* | -0.08 |
| Heart rate, bpm | 0.22† | -0.001 | -0.01 |
| Total cholesterol, mmol/L | 0.09 | -0.07 | 0.01 |
| Triglycerides, mmol/L | 0.15* | -0.20 | -0.12 |
| HDL cholesterol, mmol/L | -0.27† | 0.08 | 0.11 |
| LDL cholesterol, mmol/L | 0.17* | -0.07 | -0.04 |
| Glucose, mmol/L | 0.03 | -0.07 | -0.11 |
| Hemoglobin A1c, % | 0.30† | -0.08 | -0.08 |
| BUN, mmol/L | 0.06 | -0.08 | -0.08 |
| Creatinine, umol/L | 0.15* | -0.23† | -0.16* |
| eGFR, ml/min/1.73 m^2^ | -0.11 | 0.15* | 0.01 |
| hs-CRP, mg/dL | 0.24* | -0.13 | -0.01 |
| Smoking, pack-years | 0.23* | 0.02 | -0.11 |
| White blood cells, × 10^3^/μL | - | -0.03 | -0.05 |
| Neutrophils, × 10^3^/μL | 0.88† | 0.03 | -0.05 |
| Lymphocytes, × 10^3^/μL | 0.62† | -0.15 | -0.03 |
| Monocytes, × 10^3^/μL | 0.65† | 0.01 | -0.06 |
| Eosinophils, × 10^3^/μL | 0.36† | 0.08 | -0.01 |
| Basophils, × 10^3^/μL | 0.29† | -0.14 | -0.09 |
| Baseline BAD, mm | 0.16* | -0.55† | -0.51† |
| FMD, % | -0.03 | - | 0.42† |
| NID, % | -0.05 | 0.42 | - |

*P<0.05, †P<0.01

FMD indicates flow-mediated vasodilation; NID, nitroglycerine-induced vasodilation; BAD, brachial artery diameter; HDL, high-density lipoprotein; LDL, low-density lipoprotein; BUN, blood urea nitrogen; eGFR, estimated-glomerular filtration rate; hs-CRP, high-sensitive C-reactive protein.

**Supplemental Table S16.** Univariate Analysis of Relationships among White Blood Cell, FMD, NID and Variables in Subjects with 55-64 Years of Age

| Variables | White blood cell | FMD | NID |
| --- | --- | --- | --- |
| Age, yr | 0.03 | -0.03 | 0.02 |
| Body mass index, kg/m^2^ | 0.21† | -0.10 | -0.05 |
| Systolic blood pressure, mmHg | -0.03 | -0.13* | -0.10 |
| Diastolic blood pressure, mmHg | 0.05 | -0.14* | 0.01 |
| Heart rate, bpm | 0.14* | -0.05 | -0.05 |
| Total cholesterol, mmol/L | 0.08 | 0.02 | 0.13 |
| Triglycerides, mmol/L | 0.28† | -0.13* | 0.11 |
| HDL cholesterol, mmol/L | -0.20† | -0.07 | 0.06 |
| LDL cholesterol, mmol/L | 0.10 | 0.08 | 0.02 |
| Glucose, mmol/L | 0.14 | -0.14* | 0.05 |
| Hemoglobin A1c, % | 0.21† | -0.16* | 0.06 |
| BUN, mmol/L | 0.03 | 0.01 | -0.01 |
| Creatinine, umol/L | 0.14* | 0.07 | -0.01 |
| eGFR, ml/min/1.73 m^2^ | -0.03 | -0.06 | 0.04 |
| hs-CRP, mg/dL | 0.54† | 0.02 | -0.11 |
| Smoking, pack-years | 0.29† | -0.01 | -0.02 |
| White blood cells, × 10^3^/μL | - | -0.03 | 0.04 |
| Neutrophils, × 10^3^/μL | 0.87† | -0.05 | -0.02 |
| Lymphocytes, × 10^3^/μL | 0.56† | -0.05 | 0.01 |
| Monocytes, × 10^3^/μL | 0.61† | -0.02 | 0.03 |
| Eosinophils, × 10^3^/μL | 0.23† | 0.16* | 0.13 |
| Basophils, × 10^3^/μL | 0.26† | 0.06 | 0.11 |
| Baseline BAD, mm | 0.09 | -0.24† | -0.36† |
| FMD, % | -0.03 | - | 0.32† |
| NID, % | 0.04 | 0.32† | - |

*P<0.05, †P<0.01

FMD indicates flow-mediated vasodilation; NID, nitroglycerine-induced vasodilation; BAD, brachial artery diameter; HDL, high-density lipoprotein; LDL, low-density lipoprotein; BUN, blood urea nitrogen; eGFR, estimated-glomerular filtration rate; hs-CRP, high-sensitive C-reactive protein.

**Supplemental Table S17.** Univariate Analysis of Relationships among White Blood Cell, FMD, NID and Variables in Subjects with 65-74 Years of Age

| Variables | White blood cell | FMD | NID |
| --- | --- | --- | --- |
| Age, yr | -0.01 | -0.11* | -0.12* |
| Body mass index, kg/m^2^ | 0.07 | -0.02 | -0.09 |
| Systolic blood pressure, mmHg | 0.07 | 0.01 | 0.01 |
| Diastolic blood pressure, mmHg | -0.02 | 0.03 | 0.08 |
| Heart rate, bpm | 0.04 | 0.03 | -0.09 |
| Total cholesterol, mmol/L | -0.02 | -0.11* | -0.01 |
| Triglycerides, mmol/L | 0.25† | -0.02 | 0.05 |
| HDL cholesterol, mmol/L | -0.24† | -0.06 | -0.05 |
| LDL cholesterol, mmol/L | 0.05 | -0.06 | 0.05 |
| Glucose, mmol/L | 0.04 | -0.10* | 0.01 |
| Hemoglobin A1c, % | 0.05 | -0.09 | -0.08 |
| BUN, mmol/L | -0.01 | -0.06 | -0.11* |
| Creatinine, umol/L | 0.12* | -0.05 | -0.02 |
| eGFR, ml/min/1.73 m^2^ | -0.01 | 0.06 | 0.06 |
| hs-CRP, mg/dL | 0.32† | -0.09 | -0.10 |
| Smoking, pack-years | 0.15* | -0.01 | -0.01 |
| White blood cells, × 10^3^/μL | - | 0.01 | -0.01 |
| Neutrophils, × 10^3^/μL | 0.86† | 0.08 | -0.05 |
| Lymphocytes, × 10^3^/μL | 0.53† | 0.06 | 0.20† |
| Monocytes, × 10^3^/μL | 0.61† | 0.04 | -0.03 |
| Eosinophils, × 10^3^/μL | 0.23† | -0.02 | 0.08 |
| Basophils, × 10^3^/μL | 0.30† | -0.08 | 0.02 |
| Baseline BAD, mm | -0.01 | -0.31† | -0.37† |
| FMD, % | 0.01 | - | 0.40† |
| NID, % | -0.01 | 0.40† | - |

*P<0.05, †P<0.01

FMD indicates flow-mediated vasodilation; NID, nitroglycerine-induced vasodilation; BAD, brachial artery diameter; HDL, high-density lipoprotein; LDL, low-density lipoprotein; BUN, blood urea nitrogen; eGFR, estimated-glomerular filtration rate; hs-CRP, high-sensitive C-reactive protein.

**Supplemental Table S18.** Univariate Analysis of Relationships among White Blood Cell, FMD, NID and Variables in Subjects with 75 Years of Age and Older

| Variables | White blood cell | FMD | NID |
| --- | --- | --- | --- |
| Age, yr | -0.03 | -0.07 | -0.20† |
| Body mass index, kg/m^2^ | 0.05 | -0.04 | -0.20† |
| Systolic blood pressure, mmHg | 0.01 | -0.01 | -0.17* |
| Diastolic blood pressure, mmHg | 0.03 | 0.01 | -0.10 |
| Heart rate, bpm | -0.01 | -0.01 | 0.04 |
| Total cholesterol, mmol/L | 0.01 | -0.05 | 0.04 |
| Triglycerides, mmol/L | 0.18† | -0.07 | -0.08 |
| HDL cholesterol, mmol/L | -0.14* | 0.06 | 0.16* |
| LDL cholesterol, mmol/L | 0.01 | -0.09 | -0.03 |
| Glucose, mmol/L | 0.15* | -0.02 | -0.07 |
| Hemoglobin A1c, % | 0.16* | -.01 | -0.07 |
| BUN, mmol/L | 0.05 | -0.01 | -0.01 |
| Creatinine, umol/L | 0.08 | -0.08 | 0.01 |
| eGFR, ml/min/1.73 m^2^ | -0.12* | 0.07 | -0.03 |
| hs-CRP, mg/dL | 0.28* | 0.11 | 0.09 |
| Smoking, pack-years | -0.04 | 0.03 | 0.07 |
| White blood cells, × 10^3^/μL | - | -0.03 | 0.01 |
| Neutrophils, × 10^3^/μL | 0.84† | 0.01 | 0.06 |
| Lymphocytes, × 10^3^/μL | 0.51† | 0.07 | -0.04 |
| Monocytes, × 10^3^/μL | 0.54† | 0.10 | -0.18* |
| Eosinophils, × 10^3^/μL | 0.37† | -0.02 | -0.05 |
| Basophils, × 10^3^/μL | 0.31† | 0.12 | -0.13 |
| Baseline BAD, mm | 0.11 | -0.24† | -0.47† |
| FMD, % | -0.03 | - | 0.26† |
| NID, % | 0.01 | 0.26† | - |

*P<0.05, †P<0.01

FMD indicates flow-mediated vasodilation; NID, nitroglycerine-induced vasodilation; BAD, brachial artery diameter; HDL, high-density lipoprotein; LDL, low-density lipoprotein; BUN, blood urea nitrogen; eGFR, estimated-glomerular filtration rate; hs-CRP, high-sensitive C-reactive protein.

**Supplemental Table S19.** Univariate Analysis of Relationships among White Blood Cell, FMD, NID and Variables in Men

| Variables | White blood cell | FMD | NID |
| --- | --- | --- | --- |
| Age, yr | -0.19† | -0.23† | -0.25† |
| Body mass index, kg/m^2^ | 0.20† | -0.05 | -0.04 |
| Systolic blood pressure, mmHg | 0.04 | -0.15† | -0.18† |
| Diastolic blood pressure, mmHg | 0.06 | -0.07* | -0.01 |
| Heart rate, bpm | 0.12† | 0.01 | -0.01 |
| Total cholesterol, mmol/L | 0.14† | -0.04 | 0.11† |
| Triglycerides, mmol/L | 0.17† | -0.08* | 0.05 |
| HDL cholesterol, mmol/L | -0.19† | -0.04 | 0.01 |
| LDL cholesterol, mmol/L | 0.16† | 0.01 | 0.07 |
| Glucose, mmol/L | -0.02 | -0.16† | -0.04 |
| Hemoglobin A1c, % | 0.11* | -0.15† | -0.09* |
| BUN, mmol/L | -0.03 | -0.07 | -0.12† |
| Creatinine, umol/L | 0.05 | -0.05 | -0.11† |
| eGFR, ml/min/1.73 m^2^ | 0.05 | 0.11† | 0.15† |
| hs-CRP, mg/dL | 0.19† | 0.09 | -0.04 |
| Smoking, pack-years | 0.12† | -0.02 | -0.09* |
| White blood cells, × 10^3^/μL | - | 0.01 | 0.05 |
| Neutrophils, × 10^3^/μL | 0.87† | 0.01 | -0.01 |
| Lymphocytes, × 10^3^/μL | 0.52† | 0.01 | 0.08 |
| Monocytes, × 10^3^/μL | 0.58† | 0.04 | -0.01 |
| Eosinophils, × 10^3^/μL | 0.27† | 0.07 | 0.06 |
| Basophils, × 10^3^/μL | 0.26† | 0.04 | -0.02 |
| Baseline BAD, mm | -0.05 | -0.36† | -0.45† |
| FMD, % | 0.01 | - | 0.39† |
| NID, % | 0.05 | 0.39† | - |

*P<0.05, †P<0.01

FMD indicates flow-mediated vasodilation; NID, nitroglycerine-induced vasodilation HDL, high-density lipoprotein; LDL, low-density lipoprotein; BUN, blood urea nitrogen; eGFR, estimated-glomerular filtration rate; hs-CRP, high-sensitive C-reactive protein; BAD, brachial artery diameter.

**Supplemental Table S20.** Univariate Analysis of Relationships among White Blood Cell, FMD, NID and Variables in Women

| Variables | White blood cell | FMD | NID |
| --- | --- | --- | --- |
| Age, yr | -0.03 | -0.30† | -0.32† |
| Body mass index, kg/m^2^ | 0.18† | 0.08 | 0.04 |
| Systolic blood pressure, mmHg | 0.08 | -0.04 | 0.01 |
| Diastolic blood pressure, mmHg | 0.10* | 0.06 | 0.17† |
| Heart rate, bpm | 0.06 | 0.04 | 0.02 |
| Total cholesterol, mmol/L | 0.06 | -0.02 | 0.09 |
| Triglycerides, mmol/L | 0.22† | -0.01 | 0.06 |
| HDL cholesterol, mmol/L | -0.19† | 0.01 | 0.01 |
| LDL cholesterol, mmol/L | 0.07 | -0.05 | 0.06 |
| Glucose, mmol/L | 0.11* | -0.10* | -0.02 |
| Hemoglobin A1c, % | 0.15† | -0.09 | -0.09 |
| BUN, mmol/L | 0.03 | -0.09* | -0.17† |
| Creatinine, umol/L | 0.12† | -0.09* | -0.10* |
| eGFR, ml/min/1.73 m^2^ | -0.04 | 0.18† | 0.17† |
| hs-CRP, mg/dL | 0.08 | -0.01 | 0.01 |
| Smoking, pack-years | 0.06 | -0.20* | -0.03 |
| White blood cells, × 10^3^/μL | - | 0.02 | 0.01 |
| Neutrophils, × 10^3^/μL | 0.89† | 0.07 | 0.04 |
| Lymphocytes, × 10^3^/μL | 0.57† | 0.03 | 0.07 |
| Monocytes, × 10^3^/μL | 0.58† | 0.06 | -0.06 |
| Eosinophils, × 10^3^/μL | 0.24† | 0.05 | 0.08 |
| Basophils, × 10^3^/μL | 0.19† | 0.07 | -0.06 |
| Baseline BAD, mm | 0.07 | -0.37† | -0.50† |
| FMD, % | 0.02 | - | 0.42† |
| NID, % | 0.01 | 0.42† | - |

*P<0.05, †P<0.01

FMD indicates flow-mediated vasodilation; NID, nitroglycerine-induced vasodilation HDL, high-density lipoprotein; LDL, low-density lipoprotein; BUN, blood urea nitrogen; eGFR, estimated-glomerular filtration rate; hs-CRP, high-sensitive C-reactive protein; BAD, brachial artery diameter.

**Supplemental Table S21.** Univariate Analysis of Relationships among White Blood Cell, FMD, NID and Variables in Subjects with Body Mass Index under 25 kg/m^2^

| Variables | White blood cell | FMD | NID |
| --- | --- | --- | --- |
| Age, yr | -0.04 | -0.25† | -0.26† |
| Body mass index, kg/m^2^ | 0.14† | 0.02 | -0.03 |
| Systolic blood pressure, mmHg | 0.07* | -0.12 | -0.09* |
| Diastolic blood pressure, mmHg | 0.07* | -0.02 | 0.08* |
| Heart rate, bpm | 0.04 | 0.01 | -0.06 |
| Total cholesterol, mmol/L | 0.03 | -0.02 | 0.10* |
| Triglycerides, mmol/L | 0.21† | -0.06 | 0.05 |
| HDL cholesterol, mmol/L | -0.20† | -0.01 | 0.01 |
| LDL cholesterol, mmol/L | 0.05 | -0.01 | 0.09* |
| Glucose, mmol/L | 0.05 | -0.11† | -0.02 |
| Hemoglobin A1c, % | 0.12† | -0.09* | -0.10* |
| BUN, mmol/L | 0.02 | -0.06 | -0.15† |
| Creatinine, umol/L | 0.14† | -0.02 | -0.10† |
| eGFR, ml/min/1.73 m^2^ | -0.03 | 0.10† | 0.15† |
| hs-CRP, mg/dL | 0.15* | 0.06 | -0.01 |
| Smoking, pack-years | 0.18† | -0.06 | -0.06 |
| White blood cells, × 10^3^/μL | - | 0.13 | 0.04 |
| Neutrophils, × 10^3^/μL | 0.89† | 0.04 | 0.01 |
| Lymphocytes, × 10^3^/μL | 0.50† | 0.06 | 0.11* |
| Monocytes, × 10^3^/μL | 0.56† | 0.06 | -0.06 |
| Eosinophils, × 10^3^/μL | 0.27† | 0.04 | 0.04 |
| Basophils, × 10^3^/μL | 0.24† | 0.09* | -0.05 |
| Baseline BAD, mm | 0.13† | -0.40† | -0.40† |
| FMD, % | 0.03 | - | 0.38† |
| NID, % | 0.04 | 0.38† | - |

*P<0.05, †P<0.01

FMD indicates flow-mediated vasodilation; NID, nitroglycerine-induced vasodilation HDL, high-density lipoprotein; LDL, low-density lipoprotein; BUN, blood urea nitrogen; eGFR, estimated-glomerular filtration rate; hs-CRP, high-sensitive C-reactive protein; BAD, brachial artery diameter.

**Supplemental Table S22.** Univariate Analysis of Relationships among White Blood Cell, FMD, NID and Variables in Subjects with Body Mass Index over 25 kg/m^2^

| Variables | White blood cell | FMD | NID |
| --- | --- | --- | --- |
| Age, yr | -0.25† | -0.28† | -0.32† |
| Body mass index, kg/m^2^ | 0.17† | 0.06 | 0.03 |
| Systolic blood pressure, mmHg | -0.01 | -0.04 | -0.12* |
| Diastolic blood pressure, mmHg | 0.06 | -0.01 | 0.03 |
| Heart rate, bpm | 0.11* | 0.07 | 0.11* |
| Total cholesterol, mmol/L | 0.17† | -0.03 | 0.11* |
| Triglycerides, mmol/L | 0.13† | -0.06 | 0.05 |
| HDL cholesterol, mmol/L | -0.14† | -0.02 | 0.04 |
| LDL cholesterol, mmol/L | 0.19† | -0.01 | 0.03 |
| Glucose, mmol/L | -0.01 | -0.17† | -0.05 |
| Hemoglobin A1c, % | 0.09 | -0.17† | -0.08 |
| BUN, mmol/L | -0.01 | -0.13† | -0.11* |
| Creatinine, umol/L | 0.10* | -0.20† | -0.11* |
| eGFR, ml/min/1.73 m^2^ | 0.10* | 0.22† | 0.17† |
| hs-CRP, mg/dL | 0.18* | 0.06 | -0.07 |
| Smoking, pack-years | -0.01 | -0.12* | -0.17† |
| White blood cells, × 10^3^/μL | - | -0.05 | 0.01 |
| Neutrophils, × 10^3^/μL | 0.86 | -0.01 | -0.01 |
| Lymphocytes, × 10^3^/μL | 0.56† | -0.04 | 0.04 |
| Monocytes, × 10^3^/μL | 0.59† | -0.05 | -0.03 |
| Eosinophils, × 10^3^/μL | 0.26† | 0.04 | 0.04 |
| Basophils, × 10^3^/μL | 0.25† | -0.04 | -0.04 |
| Baseline BAD, mm | 0.03 | -0.29† | -0.40† |
| FMD, % | -0.05 | - | 0.44† |
| NID, % | 0.01 | 0.44† | - |

*P<0.05, †P<0.01

FMD indicates flow-mediated vasodilation; NID, nitroglycerine-induced vasodilation HDL, high-density lipoprotein; LDL, low-density lipoprotein; BUN, blood urea nitrogen; eGFR, estimated-glomerular filtration rate; hs-CRP, high-sensitive C-reactive protein; BAD, brachial artery diameter.

**Supplemental Table S23.** Univariate Analysis of Relationships among White Blood Cell, FMD, NID and Variables in Subjects without Hypertension

| Variables | White blood cell | FMD | NID |
| --- | --- | --- | --- |
| Age, yr | -0.12* | -0.36† | -0.20† |
| Body mass index, kg/m^2^ | 0.39† | 0.05 | 0.13* |
| Systolic blood pressure, mmHg | 0.02 | -0.07 | -0.05 |
| Diastolic blood pressure, mmHg | 0.06 | 0.03 | 0.06 |
| Heart rate, bpm | -0.01 | -0.02 | -0.01 |
| Total cholesterol, mmol/L | 0.09 | -0.03 | 0.14* |
| Triglycerides, mmol/L | 0.23† | -0.09 | 0.10 |
| HDL cholesterol, mmol/L | -0.17† | 0.06 | -0.09 |
| LDL cholesterol, mmol/L | 0.10 | -0.02 | 0.16* |
| Glucose, mmol/L | 0.03 | -0.11 | -0.14* |
| Hemoglobin A1c, % | 0.08 | -0.06 | -0.07 |
| BUN, mmol/L | -0.06 | -0.10 | -0.10 |
| Creatinine, umol/L | 0.24† | -0.10 | 0.06 |
| eGFR, ml/min/1.73 m^2^ | 0.05 | 0.21† | 0.05 |
| hs-CRP, mg/dL | -0.08 | -0.05 | 0.07 |
| Smoking, pack-years | 0.14 | -0.19* | -0.19* |
| White blood cells, × 10^3^/μL | - | -0.03 | 0.09 |
| Neutrophils, × 10^3^/μL | 0.89† | -0.04 | 0.06 |
| Lymphocytes, × 10^3^/μL | 0.54† | 0.05 | 0.21† |
| Monocytes, × 10^3^/μL | 0.59† | 0.01 | -0.01 |
| Eosinophils, × 10^3^/μL | 0.32† | 0.07 | 0.16* |
| Basophils, × 10^3^/μL | 0.22† | -0.01 | -0.11 |
| Baseline BAD, mm | 0.11 | -0.32† | -0.32† |
| FMD, % | -0.03 | - | 0.39† |
| NID, % | 0.09 | 0.39† | - |

*P<0.05, †P<0.01

FMD indicates flow-mediated vasodilation; NID, nitroglycerine-induced vasodilation HDL, high-density lipoprotein; LDL, low-density lipoprotein; BUN, blood urea nitrogen; eGFR, estimated-glomerular filtration rate; hs-CRP, high-sensitive C-reactive protein; BAD, brachial artery diameter.

**Supplemental Table S24.** Univariate Analysis of Relationships among White Blood Cell, FMD, NID and Variables in Subjects with Hypertension

| Variables | White blood cell | FMD | NID |
| --- | --- | --- | --- |
| Age, yr | -0.14† | -0.20 | -0.26† |
| Body mass index, kg/m^2^ | 0.16† | 0.04 | 0.02 |
| Systolic blood pressure, mmHg | 0.05 | -0.05 | -0.04 |
| Diastolic blood pressure, mmHg | 0.08† | 0.02 | 0.13† |
| Heart rate, bpm | 0.10† | 0.04 | 0.02 |
| Total cholesterol, mmol/L | 0.08* | -0.03 | 0.08* |
| Triglycerides, mmol/L | 0.19† | -0.05 | 0.06 |
| HDL cholesterol, mmol/L | -0.22† | -0.03 | 0.03 |
| LDL cholesterol, mmol/L | 0.11† | -0.02 | 0.02 |
| Glucose, mmol/L | 0.04 | -0.15† | -0.01 |
| Hemoglobin A1c, % | 0.14† | -0.14† | -0.09* |
| BUN, mmol/L | 0.02 | -0.07* | -0.15† |
| Creatinine, umol/L | 0.11† | -0.07* | -0.12† |
| eGFR, ml/min/1.73 m^2^ | 0.02 | 0.10† | 0.16† |
| hs-CRP, mg/dL | 0.20† | 0.10 | -0.04 |
| Smoking, pack-years | 0.09* | -0.05 | -0.08 |
| White blood cells, × 10^3^/μL | - | 0.01 | 0.01 |
| Neutrophils, × 10^3^/μL | 0.87† | 0.05 | -0.01 |
| Lymphocytes, × 10^3^/μL | 0.54† | 0.01 | 0.05 |
| Monocytes, × 10^3^/μL | 0.59† | 0.02 | -0.06 |
| Eosinophils, × 10^3^/μL | 0.26† | 0.04 | 0.02 |
| Basophils, × 10^3^/μL | 0.26† | 0.05 | -0.01 |
| Baseline BAD, mm | 0.06* | -0.32† | -0.40† |
| FMD, % | 0.01 | - | 0.38† |
| NID, % | 0.01 | 0.38† | - |

*P<0.05, †P<0.01

FMD indicates flow-mediated vasodilation; NID, nitroglycerine-induced vasodilation HDL, high-density lipoprotein; LDL, low-density lipoprotein; BUN, blood urea nitrogen; eGFR, estimated-glomerular filtration rate; hs-CRP, high-sensitive C-reactive protein; BAD, brachial artery diameter.

**Supplemental Table S25.** Univariate Analysis of Relationships among White Blood Cell, FMD, NID and Variables in Subjects without Dyslipidemia

| Variables | White blood cell | FMD | NID |
| --- | --- | --- | --- |
| Age, yr | -0.11* | -0.31† | -0.28† |
| Body mass index, kg/m^2^ | 0.11* | 0.05 | -0.02 |
| Systolic blood pressure, mmHg | 0.05 | -0.10* | -0.11* |
| Diastolic blood pressure, mmHg | 0.06 | -0.10* | -0.01 |
| Heart rate, bpm | 0.05 | -0.02 | 0.01 |
| Total cholesterol, mmol/L | 0.02 | -0.06 | 0.06 |
| Triglycerides, mmol/L | 0.17† | -0.04 | 0.07 |
| HDL cholesterol, mmol/L | -0.19† | -0.05 | 0.01 |
| LDL cholesterol, mmol/L | 0.06 | -0.01 | 0.02 |
| Glucose, mmol/L | 0.03 | -0.09 | -0.05 |
| Hemoglobin A1c, % | 0.13* | -0.09 | -0.05 |
| BUN, mmol/L | -0.02 | -0.15† | -0.18† |
| Creatinine, umol/L | 0.06 | -0.14† | -0.16† |
| eGFR, ml/min/1.73 m^2^ | 0.02 | 0.24† | 0.19† |
| hs-CRP, mg/dL | 0.05 | -0.09 | -0.07 |
| Smoking, pack-years | 0.10 | -0.08 | -0.03 |
| White blood cells, × 10^3^/μL | - | 0.03 | 0.01 |
| Neutrophils, × 10^3^/μL | 0.88† | -0.01 | -0.07 |
| Lymphocytes, × 10^3^/μL | 0.52† | 0.07 | 0.08 |
| Monocytes, × 10^3^/μL | 0.59† | -0.01 | -0.07 |
| Eosinophils, × 10^3^/μL | 0.29† | 0.08 | 0.03 |
| Basophils, × 10^3^/μL | 0.28† | 0.04 | -0.07 |
| Baseline BAD, mm | 0.05 | -0.36† | -0.38† |
| FMD, % | 0.03 | - | 0.41† |
| NID, % | 0.01 | 0.41† | - |

*P<0.05, †P<0.01

FMD indicates flow-mediated vasodilation; NID, nitroglycerine-induced vasodilation HDL, high-density lipoprotein; LDL, low-density lipoprotein; BUN, blood urea nitrogen; eGFR, estimated-glomerular filtration rate; hs-CRP, high-sensitive C-reactive protein; BAD, brachial artery diameter.

**Supplemental Table S26.** Univariate Analysis of Relationships among White Blood Cell, FMD, NID and Variables in Subjects with Dyslipidemia

| Variables | White blood cell | FMD | NID |
| --- | --- | --- | --- |
| Age, yr | -0.18† | -0.21† | -0.28† |
| Body mass index, kg/m^2^ | 0.25† | -0.02 | 0.01 |
| Systolic blood pressure, mmHg | 0.06 | -0.10† | -0.09* |
| Diastolic blood pressure, mmHg | 0.10† | 0.03 | -0.09* |
| Heart rate, bpm | 0.09† | 0.05 | 0.01 |
| Total cholesterol, mmol/L | 0.09* | -0.01 | 0.12† |
| Triglycerides, mmol/L | 0.20† | -0.07* | 0.05 |
| HDL cholesterol, mmol/L | -0.21† | 0.02 | 0.01 |
| LDL cholesterol, mmol/L | 0.12† | -0.01 | -0.10* |
| Glucose, mmol/L | 0.03 | -0.16† | -0.03 |
| Hemoglobin A1c, % | 0.11† | -0.13† | -0.09* |
| BUN, mmol/L | 0.01 | -0.05 | -0.12† |
| Creatinine, umol/L | 0.17† | -0.05 | -0.07 |
| eGFR, ml/min/1.73 m^2^ | 0.04 | 0.06 | 0.13† |
| hs-CRP, mg/dL | 0.19† | 0.11 | -0.01 |
| Smoking, pack-years | 0.11* | -0.08 | -0.12* |
| White blood cells, × 10^3^/μL | - | -0.02 | 0.05 |
| Neutrophils, × 10^3^/μL | 0.88† | 0.05 | 0.04 |
| Lymphocytes, × 10^3^/μL | 0.54† | -0.02 | 0.08 |
| Monocytes, × 10^3^/μL | 0.58† | 0.04 | -0.03 |
| Eosinophils, × 10^3^/μL | 0.27† | 0.02 | 0.06 |
| Basophils, × 10^3^/μL | 0.23† | 0.04 | -0.02 |
| Baseline BAD, mm | 0.09† | -0.33† | -0.41† |
| FMD, % | -0.02 | - | 0.40† |
| NID, % | 0.05 | 0.40† | - |

*P<0.05, †P<0.01

FMD indicates flow-mediated vasodilation; NID, nitroglycerine-induced vasodilation HDL, high-density lipoprotein; LDL, low-density lipoprotein; BUN, blood urea nitrogen; eGFR, estimated-glomerular filtration rate; hs-CRP, high-sensitive C-reactive protein; BAD, brachial artery diameter.

**Supplemental Table S27.** Univariate Analysis of Relationships among White Blood Cell, FMD, NID and Variables in Subjects without Diabetes Mellitus

| Variables | White blood cell | FMD | NID |
| --- | --- | --- | --- |
| Age, yr | -0.13† | -0.25† | -0.23† |
| Body mass index, kg/m^2^ | 0.19† | 0.02 | -0.03 |
| Systolic blood pressure, mmHg | 0.08* | -0.10† | -0.10† |
| Diastolic blood pressure, mmHg | 0.12† | -0.05 | 0.01 |
| Heart rate, bpm | 0.11† | 0.04 | 0.01 |
| Total cholesterol, mmol/L | 0.12† | -0.01 | 0.07 |
| Triglycerides, mmol/L | 0.21† | -0.03 | 0.03 |
| HDL cholesterol, mmol/L | -0.19† | 0.01 | 0.01 |
| LDL cholesterol, mmol/L | 0.12† | -0.02 | 0.04 |
| Glucose, mmol/L | 0.03 | -0.09* | -0.06 |
| Hemoglobin A1c, % | 0.07 | -0.06 | -0.06 |
| BUN, mmol/L | -0.05 | -0.07 | -0.14† |
| Creatinine, umol/L | 0.09† | -0.10† | -0.09* |
| eGFR, ml/min/1.73 m^2^ | 0.04 | 0.15† | 0.13† |
| hs-CRP, mg/dL | 0.14* | 0.05 | 0.02 |
| Smoking, pack-years | 0.09* | -0.10* | -0.11* |
| White blood cells, × 10^3^/μL | - | -0.01 | 0.04 |
| Neutrophils, × 10^3^/μL | 0.87† | 0.04 | 0.01 |
| Lymphocytes, × 10^3^/μL | 0.54† | 0.04 | 0.08* |
| Monocytes, × 10^3^/μL | 0.59† | 0.03 | -0.02 |
| Eosinophils, × 10^3^/μL | 0.26† | 0.05 | 0.05 |
| Basophils, × 10^3^/μL | 0.28† | 0.01 | -0.05 |
| Baseline BAD, mm | 0.11† | -0.37† | -0.40† |
| FMD, % | -0.01 | - | 0.45† |
| NID, % | 0.04 | 0.45† | - |

*P<0.05, †P<0.01

FMD indicates flow-mediated vasodilation; NID, nitroglycerine-induced vasodilation HDL, high-density lipoprotein; LDL, low-density lipoprotein; BUN, blood urea nitrogen; eGFR, estimated-glomerular filtration rate; hs-CRP, high-sensitive C-reactive protein; BAD, brachial artery diameter.

**Supplemental Table S28.** Univariate Analysis of Relationships among White Blood Cell, FMD, NID and Variables in Subjects with Diabetes Mellitus

| Variables | White blood cell | FMD | NID |
| --- | --- | --- | --- |
| Age, yr | -0.25† | -0.19† | -0.35† |
| Body mass index, kg/m^2^ | 0.22† | 0.02 | 0.08 |
| Systolic blood pressure, mmHg | 0.01 | -0.09* | -0.09 |
| Diastolic blood pressure, mmHg | 0.06 | 0.01 | 0.14† |
| Heart rate, bpm | 0.01 | 0.01 | 0.01 |
| Total cholesterol, mmol/L | 0.07 | -0.12* | 0.12 |
| Triglycerides, mmol/L | 0.16† | -0.10* | 0.10 |
| HDL cholesterol, mmol/L | -0.22† | -0.07 | -0.01 |
| LDL cholesterol, mmol/L | 0.12* | -0.06 | 0.08 |
| Glucose, mmol/L | 0.04 | -0.13* | 0.07 |
| Hemoglobin A1c, % | 0.08 | -0.05 | -0.03 |
| BUN, mmol/L | 0.04 | -0.06 | -0.11* |
| Creatinine, umol/L | 0.16† | -0.03 | -0.10 |
| eGFR, ml/min/1.73 m^2^ | 0.01 | 0.06 | 0.17† |
| hs-CRP, mg/dL | 0.26* | 0.10 | -0.22* |
| Smoking, pack-years | 0.08 | 0.04 | -0.05 |
| White blood cells, × 10^3^/μL | - | 0.04 | 0.05 |
| Neutrophils, × 10^3^/μL | 0.89† | 0.05 | 0.01 |
| Lymphocytes, × 10^3^/μL | 053† | -0.2 | 0.09 |
| Monocytes, × 10^3^/μL | 0.55† | 0.04 | -0.07 |
| Eosinophils, × 10^3^/μL | 0.29† | 0.07 | 0.08 |
| Basophils, × 10^3^/μL | 0.16† | 0.12* | -0.01 |
| Baseline BAD, mm | -0.02 | -0.27† | -0.39† |
| FMD, % | 0.04 | - | 0.04† |
| NID, % | 0.05 | 0.26† | - |

*P<0.05, †P<0.01

FMD indicates flow-mediated vasodilation; NID, nitroglycerine-induced vasodilation HDL, high-density lipoprotein; LDL, low-density lipoprotein; BUN, blood urea nitrogen; eGFR, estimated-glomerular filtration rate; hs-CRP, high-sensitive C-reactive protein; BAD, brachial artery diameter.

**Supplemental Table S29.** Univariate Analysis of Relationships among White Blood Cell, FMD, NID and Variables in Non-smoker

| Variables | White blood cell | FMD | NID |
| --- | --- | --- | --- |
| Age, yr | -0.09† | -0.28† | -0.31† |
| Body mass index, kg/m^2^ | 0.22† | 0.02 | -0.02 |
| Systolic blood pressure, mmHg | 0.05 | -0.09† | -0.07* |
| Diastolic blood pressure, mmHg | 0.07* | 0.01 | 0.09† |
| Heart rate, bpm | 0.07* | 0.05 | -0.01 |
| Total cholesterol, mmol/L | 0.07* | -0.01 | 0.11† |
| Triglycerides, mmol/L | 0.20† | 0.07* | 0.05 |
| HDL cholesterol, mmol/L | -0.21† | 0.01 | 0.04 |
| LDL cholesterol, mmol/L | 0.10† | -0.02 | 0.06 |
| Glucose, mmol/L | 0.05 | -0.15† | -0.05 |
| Hemoglobin A1c, % | 0.12† | -0.14† | -0.11† |
| BUN, mmol/L | 0.04 | -0.09† | -0.13† |
| Creatinine, umol/L | 0.14† | -0.09† | -0.10† |
| eGFR, ml/min/1.73 m^2^ | -0.04 | 0.16† | 0.17† |
| hs-CRP, mg/dL | 0.20† | 0.07 | -0.02 |
| Smoking, pack-years | 0.11* | -0.10* | -0.13† |
| White blood cells, × 10^3^/μL | - | 0.01 | 0.02 |
| Neutrophils, × 10^3^/μL | 0.87† | 0.03 | -0.01 |
| Lymphocytes, × 10^3^/μL | 0.53† | 0.02 | 0.07 |
| Monocytes, × 10^3^/μL | 0.59† | 0.04 | -0.06 |
| Eosinophils, × 10^3^/μL | 0.28† | 0.02 | 0.05 |
| Basophils, × 10^3^/μL | 0.23† | 0.03 | -0.04 |
| Baseline BAD, mm | 0.09† | -0.34† | -0.42† |
| FMD, % | 0.01 | - | 0.41† |
| NID, % | 0.02 | 0.41† | - |

*P<0.05, †P<0.01

FMD indicates flow-mediated vasodilation; NID, nitroglycerine-induced vasodilation HDL, high-density lipoprotein; LDL, low-density lipoprotein; BUN, blood urea nitrogen; eGFR, estimated-glomerular filtration rate; hs-CRP, high-sensitive C-reactive protein; BAD, brachial artery diameter.

**Supplemental Table S30.** Univariate Analysis of Relationships among White Blood Cell, FMD, NID and Variables in Current Smoker

| Variables | White blood cell | FMD | NID |
| --- | --- | --- | --- |
| Age, yr | -0.19† | -0.10 | -0.04 |
| Body mass index, kg/m^2^ | 0.15* | -0.08 | 0.03 |
| Systolic blood pressure, mmHg | 0.03 | -0.16* | -0.24 |
| Diastolic blood pressure, mmHg | 0.10 | -0.12† | -0.12 |
| Heart rate, bpm | 0.10 | -0.08 | 0.03 |
| Total cholesterol, mmol/L | 0.14* | -0.06 | 0.07 |
| Triglycerides, mmol/L | 0.10 | -0.07 | 0.04 |
| HDL cholesterol, mmol/L | -0.16* | -0.02 | -0.11 |
| LDL cholesterol, mmol/L | 0.16* | 0.05 | 0.12 |
| Glucose, mmol/L | -0.03 | -0.12 | 0.03 |
| Hemoglobin A1c, % | 0.13 | -0.09 | -0.03 |
| BUN, mmol/L | 0.03 | -0.06 | -0.15* |
| Creatinine, umol/L | 0.13 | -0.08 | -0.10 |
| eGFR, ml/min/1.73 m^2^ | 0.05 | 0.03 | 0.08 |
| hs-CRP, mg/dL | 0.07 | -0.05 | -0.13 |
| Smoking, pack-years | 0.08 | -0.03 | -0.01 |
| White blood cells, × 10^3^/μL | - | -0.05 | 0.01 |
| Neutrophils, × 10^3^/μL | 0.89† | 0.01 | -0.03 |
| Lymphocytes, × 10^3^/μL | 0.44† | -0.04 | 0.10 |
| Monocytes, × 10^3^/μL | 0.51† | -0.10 | 0.01 |
| Eosinophils, × 10^3^/μL | 0.23† | 0.14 | 0.03 |
| Basophils, × 10^3^/μL | 0.26† | 0.06 | -0.03 |
| Baseline BAD, mm | -0.01 | -0.33† | -0.30† |
| FMD, % | -0.05 | - | 0.36† |
| NID, % | 0.01 | 0.36† | - |

*P<0.05, †P<0.01

FMD indicates flow-mediated vasodilation; NID, nitroglycerine-induced vasodilation HDL, high-density lipoprotein; LDL, low-density lipoprotein; BUN, blood urea nitrogen; eGFR, estimated-glomerular filtration rate; hs-CRP, high-sensitive C-reactive protein; BAD, brachial artery diameter.

**Supplemental Table S31.** Univariate Analysis of Relationships among White Blood Cell, FMD, NID and Variables in Subjects with Low White Blood Cells Count

| Variables | White blood cell | FMD | NID |
| --- | --- | --- | --- |
| Age, yr | 0.01 | -0.33† | -0.31† |
| Body mass index, kg/m^2^ | 0.26† | 0.03 | -0.01 |
| Systolic blood pressure, mmHg | 0.13† | -0.08 | -0.01 |
| Diastolic blood pressure, mmHg | 0.09* | 0.04 | 0.20† |
| Heart rate, bpm | -0.02 | 0.04 | -0.02 |
| Total cholesterol, mmol/L | -0.07 | 0.05 | 0.19† |
| Triglycerides, mmol/L | 0.09 | -0.02 | 0.02 |
| HDL cholesterol, mmol/L | -0.10* | 0.01 | 0.02 |
| LDL cholesterol, mmol/L | -0.06 | 0.03 | 0.18† |
| Glucose, mmol/L | 0.09 | -0.14† | 0.05 |
| Hemoglobin A1c, % | 0.02 | -0.15† | -0.04 |
| BUN, mmol/L | -0.01 | -0.10* | -0.10 |
| Creatinine, umol/L | 0.18† | -0.13† | -0.10 |
| eGFR, ml/min/1.73 m^2^ | -0.06 | 0.19† | 0.16† |
| hs-CRP, mg/dL | -0.05 | -0.01 | 0.03 |
| Smoking, pack-years | -0.18† | -0.03 | -0.04 |
| White blood cells, × 10^3^/μL | - | -0.01 | 0.06 |
| Neutrophils, × 10^3^/μL | 0.69† | 0.02 | 0.09 |
| Lymphocytes, × 10^3^/μL | 0.32† | 0.09 | 0.07 |
| Monocytes, × 10^3^/μL | 0.33† | 0.02 | -0.09 |
| Eosinophils, × 10^3^/μL | 0.24† | 0.01 | 0.06 |
| Basophils, × 10^3^/μL | 0.19† | 0.08 | 0.01 |
| Baseline BAD, mm | 0.11* | -0.31† | -0.38† |
| FMD, % | -0.01 | - | 0.38† |
| NID, % | 0.06 | 0.38† | - |

*P<0.05, †P<0.01

FMD indicates flow-mediated vasodilation; NID, nitroglycerine-induced vasodilation HDL, high-density lipoprotein; LDL, low-density lipoprotein; BUN, blood urea nitrogen; eGFR, estimated-glomerular filtration rate; hs-CRP, high-sensitive C-reactive protein; BAD, brachial artery diameter.

**Supplemental Table S32.** Univariate Analysis of Relationships among White Blood Cell, FMD, NID and Variables in Subjects with Middle White Blood Cells Count

| Variables | White blood cell | FMD | NID |
| --- | --- | --- | --- |
| Age, yr | -0.02 | -0.22† | -0.26† |
| Body mass index, kg/m^2^ | 0.10* | 0.03 | -0.05 |
| Systolic blood pressure, mmHg | 0.07 | -0.12† | -0.06 |
| Diastolic blood pressure, mmHg | -0.01 | -0.11* | 0.03 |
| Heart rate, bpm | 0.04 | -0.04 | -0.01 |
| Total cholesterol, mmol/L | 0.01 | -0.05 | 0.11* |
| Triglycerides, mmol/L | 0.09 | -0.07 | 0.13* |
| HDL cholesterol, mmol/L | -0.08 | -0.03 | 0.01 |
| LDL cholesterol, mmol/L | -0.01 | -0.04 | 0.06 |
| Glucose, mmol/L | 0.02 | -0.15† | -0.04 |
| Hemoglobin A1c, % | -0.01 | -0.09 | -0.09 |
| BUN, mmol/L | -0.02 | -0.07 | -0.16† |
| Creatinine, umol/L | -0.03 | -0.09 | -0.14† |
| eGFR, ml/min/1.73 m^2^ | 0.05 | 0.14† | 0.18† |
| hs-CRP, mg/dL | -0.04 | -0.01 | -0.01 |
| Smoking, pack-years | 0.11 | -0.17† | -0.16* |
| White blood cells, × 10^3^/μL | - | 0.06 | -0.01 |
| Neutrophils, × 10^3^/μL | 0.38† | 0.02 | -0.08 |
| Lymphocytes, × 10^3^/μL | 0.27† | 0.06 | 0.15† |
| Monocytes, × 10^3^/μL | 0.20† | -0.01 | -0.13* |
| Eosinophils, × 10^3^/μL | 0.05 | 0.03 | 0.04 |
| Basophils, × 10^3^/μL | 0.08 | 0.08 | -0.11 |
| Baseline BAD, mm | -0.06 | -0.35† | -0.40† |
| FMD, % | 0.06 | - | 0.42† |
| NID, % | -0.01 | 0.42† | - |

*P<0.05, †P<0.01

FMD indicates flow-mediated vasodilation; NID, nitroglycerine-induced vasodilation HDL, high-density lipoprotein; LDL, low-density lipoprotein; BUN, blood urea nitrogen; eGFR, estimated-glomerular filtration rate; hs-CRP, high-sensitive C-reactive protein; BAD, brachial artery diameter.

**Supplemental Table S33.** Univariate Analysis of Relationships among White Blood Cell, FMD, NID and Variables in Subjects with High White Blood Cells Count

| Variables | White blood cell | FMD | NID |
| --- | --- | --- | --- |
| Age, yr | -0.13† | -0.22† | -0.26† |
| Body mass index, kg/m^2^ | 0.11* | -0.05 | 0.03 |
| Systolic blood pressure, mmHg | -0.07 | -0.10* | -0.23† |
| Diastolic blood pressure, mmHg | 0.01 | 0.01 | -0.05 |
| Heart rate, bpm | 0.12* | 0.06 | 0.03 |
| Total cholesterol, mmol/L | 0.06 | -0.07 | 0.02 |
| Triglycerides, mmol/L | 0.08 | -0.09 | -0.01 |
| HDL cholesterol, mmol/L | -0.11* | 0.02 | 0.01 |
| LDL cholesterol, mmol/L | 0.10 | -0.03 | -0.02 |
| Glucose, mmol/L | -0.03 | -0.12* | -0.11 |
| Hemoglobin A1c, % | 0.05 | -0.15† | -0.13* |
| BUN, mmol/L | 0.01 | -0.08 | -0.15† |
| Creatinine, umol/L | 0.08 | -0.05 | -0.08 |
| eGFR, ml/min/1.73 m^2^ | 0.04 | 0.10* | 0.12* |
| hs-CRP, mg/dL | 0.01 | 0.10 | -0.06 |
| Smoking, pack-years | 0.17† | -0.05 | -0.08 |
| White blood cells, × 10^3^/μL | - | -0.03 | 0.05 |
| Neutrophils, × 10^3^/μL | 0.74† | 0.01 | -0.03 |
| Lymphocytes, × 10^3^/μL | 0.13* | -0.10 | 0.03 |
| Monocytes, × 10^3^/μL | 0.34† | 0.01 | 0.02 |
| Eosinophils, × 10^3^/μL | 0.05 | 0.07 | 0.05 |
| Basophils, × 10^3^/μL | 0.05 | -0.04 | -0.02 |
| Baseline BAD, mm | 0.05 | -0.35† | -0.44† |
| FMD, % | -0.03 | - | 0.41† |
| NID, % | 0.05 | 0.41† | - |

*P<0.05, †P<0.01

FMD indicates flow-mediated vasodilation; NID, nitroglycerine-induced vasodilation HDL, high-density lipoprotein; LDL, low-density lipoprotein; BUN, blood urea nitrogen; eGFR, estimated-glomerular filtration rate; hs-CRP, high-sensitive C-reactive protein; BAD, brachial artery diameter.

**Supplemental Table S34.** Univariate Analysis of Relationships among White Blood Cell, FMD, NID and Variables in Subjects with Low baseline BAD

| Variables | White blood cell | FMD | NID |
| --- | --- | --- | --- |
| Age, yr | -0.08 | -0.30† | -0.27† |
| Body mass index, kg/m^2^ | 0.19† | 0.08* | 0.06 |
| Systolic blood pressure, mmHg | 0.08* | -0.10† | -0.10* |
| Diastolic blood pressure, mmHg | 0.09* | 0.05 | 0.07 |
| Heart rate, bpm | 0.06 | 0.03 | -0.04 |
| Total cholesterol, mmol/L | 0.03 | -001 | 0.06 |
| Triglycerides, mmol/L | 0.24† | 0.05 | 0.08* |
| HDL cholesterol, mmol/L | -0.19† | -0.09* | -0.07 |
| LDL cholesterol, mmol/L | 0.06 | -0.01 | 0.06 |
| Glucose, mmol/L | 0.10* | -0.05 | -0.05 |
| Hemoglobin A1c, % | 0.13† | -0.14† | -0.10* |
| BUN, mmol/L | -0.05 | -0.09* | -0.12† |
| Creatinine, umol/L | 0.12† | -0.03 | 0.06 |
| eGFR, ml/min/1.73 m^2^ | 0.04 | 0.14† | 0.09* |
| hs-CRP, mg/dL | 0.21† | -0.02 | -0.07 |
| Smoking, pack-years | 0.12* | -0.17† | -0.13* |
| White blood cells, × 10^3^/μL | - | 0.03 | 0.08 |
| Neutrophils, × 10^3^/μL | 0.87† | 0.05 | 0.02 |
| Lymphocytes, × 10^3^/μL | 0.56† | 0.06 | 0.12* |
| Monocytes, × 10^3^/μL | 0.64† | 0.05 | 0.06 |
| Eosinophils, × 10^3^/μL | 0.30† | -0.001 | 0.09* |
| Basophils, × 10^3^/μL | 0.34† | -0.03 | -0.06 |
| Baseline BAD, mm | 0.07 | -0.14† | -0.19† |
| FMD, % | 0.03 | - | 0.36† |
| NID, % | 0.08 | 0.36† | - |

*P<0.05, †P<0.01

FMD indicates flow-mediated vasodilation; NID, nitroglycerine-induced vasodilation; BAD, brachial artery diameter; HDL, high-density lipoprotein; LDL, low-density lipoprotein; BUN, blood urea nitrogen; eGFR, estimated-glomerular filtration rate; hs-CRP, high-sensitive C-reactive protein.

**Supplemental Table S35.** Univariate Analysis of Relationships among White Blood Cell, FMD, NID and Variables in Subjects with High baseline BAD

| Variables | White blood cell | FMD | NID |
| --- | --- | --- | --- |
| Age, yr | -0.19† | -0.15† | -0.30† |
| Body mass index, kg/m^2^ | 0.18† | 0.04 | 0.08 |
| Systolic blood pressure, mmHg | 0.04 | -0.03 | -0.08 |
| Diastolic blood pressure, mmHg | 0.07 | -0.001 | 0.14† |
| Heart rate, bpm | 0.11† | 0.001 | 0.03 |
| Total cholesterol, mmol/L | 0.1 | -0.08 | 0.10* |
| Triglycerides, mmol/L | 0.25† | -0.10* | 0.10* |
| HDL cholesterol, mmol/L | -0.21† | -0.03 | -0.01 |
| LDL cholesterol, mmol/L | 0.17† | -0.02 | 0.05 |
| Glucose, mmol/L | 0.04 | -0.12† | 0.06 |
| Hemoglobin A1c, % | 0.11* | -0.13† | -0.08 |
| BUN, mmol/L | -0.01 | -0.05 | -0.10* |
| Creatinine, umol/L | 0.05 | -0.02 | -0.02 |
| eGFR, ml/min/1.73 m^2^ | 0.01 | 0.15† | 0.20† |
| hs-CRP, mg/dL | 0.43† | -0.03 | -0.01 |
| Smoking, pack-years | 0.11* | 0.02 | 0.04 |
| White blood cells, × 10^3^/μL | - | 0.01 | 0.03 |
| Neutrophils, × 10^3^/μL | 0.85† | 0.02 | -0.03 |
| Lymphocytes, × 10^3^/μL | 0.56† | 0.01 | 0.09 |
| Monocytes, × 10^3^/μL | 0.56† | 0.07 | -0.08 |
| Eosinophils, × 10^3^/μL | 0.27† | 0.11† | 0.08 |
| Basophils, × 10^3^/μL | 0.20† | 0.07 | 0.08 |
| Baseline BAD, mm | -0.05 | -0.19† | -0.29† |
| FMD, % | 0.01 | - | 0.28† |
| NID, % | 0.03 | 0.28† | - |

*P<0.05, †P<0.01

FMD indicates flow-mediated vasodilation; NID, nitroglycerine-induced vasodilation; BAD, brachial artery diameter; HDL, high-density lipoprotein; LDL, low-density lipoprotein; BUN, blood urea nitrogen; eGFR, estimated-glomerular filtration rate; hs-CRP, high-sensitive C-reactive protein.

**Supplemental Table S36.** Multiple Linear Regression Analyses of the Relationships between FMD and Variables

| Variable | Unadjusted | | Model 1 | | Model 2 | |
| --- | --- | --- | --- | --- | --- | --- |
|  | β | P value | β | P value | β | P value |
| White blood cells, × 10^3^/μL | -0.01 | 0.89 | -0.03 | 0.34 | -0.02 | 0.47 |
| Age, yr |  |  | -0.26 | <0.01 | -0.18 | <0.01 |
| Men |  |  | -0.84 | <0.01 | 0.11 | <0.01 |
| Body mass index, kg/m^2^ |  |  |  |  | 0.08 | <0.01 |
| Hypertension |  |  |  |  | -0.11 | <0.01 |
| Dyslipidemia |  |  |  |  | 0.03 | 0.18 |
| Diabetes mellitus |  |  |  |  | -0.09 | <0.01 |
| Current smoker |  |  |  |  | -0.02 | 0.46 |
| Baseline BAD, mm |  |  |  |  | -0.40 | <0.01 |

FMD indicates flow-mediated vasodilation; BAD, brachial artery diameter.

**Supplemental Table S37.** Multiple Linear Regression Analyses of the Relationships between NID and Variables

| Variable | Unadjusted | | Model 1 | | Model 2 | |
| --- | --- | --- | --- | --- | --- | --- |
|  | β | P value | β | P value | β | P value |
| White blood cells, × 10^3^/μL | 0.03 | 0.27 | 0.001 | 0.97 | 0.004 | 0.78 |
| Age, yr |  |  | -0.28 | <0.01 | -0.18 | <0.01 |
| Men |  |  | -0.03 | 0.37 | 0.23 | <0.01 |
| Body mass index, kg/m^2^ |  |  |  |  | 0.08 | <0.01 |
| Hypertension |  |  |  |  | -0.12 | <0.01 |
| Dyslipidemia |  |  |  |  | -0.02 | 0.41 |
| Diabetes mellitus |  |  |  |  | -0.05 | 0.07 |
| Current smoker |  |  |  |  | -0.03 | 0.26 |
| Baseline BAD, mm |  |  |  |  | -0.51 | <0.01 |

NID indicates nitroglycerine-induced vasodilation; BAD, brachial artery diameter.

**Supplemental Table S38.** Multiple Linear Regression Analyses of the Relationships between Baseline BAD and White Blood Cells

| Variable | Unadjusted | | Model 1 | | Model 2 | |
| --- | --- | --- | --- | --- | --- | --- |
|  | β | P value | β | P value | β | P value |
| White blood cells, × 10^3^/μL | 0.08 | <0.01 | 0.01 | 0.74 | -0.01 | 0.78 |
| Age, yr |  |  | 0.10 | <0.01 | 0.11 | <0.01 |
| Men |  |  | 0.50 | <0.01 | 0.50 | <0.01 |
| Body mass index, kg/m^2^ |  |  |  |  | 0.16 | <0.01 |
| Hypertension |  |  |  |  | 0.06 | <0.01 |
| Dyslipidemia |  |  |  |  | 0.01 | 0.82 |
| Diabetes mellitus |  |  |  |  | -0.01 | 0.84 |
| Current smoker |  |  |  |  | -0.08 | <0.01 |

BAD indicates brachial artery diameter.

**Supplemental Table S39.** Multiple Linear Regression Analyses of the Relationships between Nitroglycerine-induced Vasodilation and Lymphocytes

| Variable | Unadjusted | | Model 1 | | Model 2 | |
| --- | --- | --- | --- | --- | --- | --- |
|  | β | P value | β | P value | β | P value |
| Lymphocytes, ×10^3^/μL | 0.08 | 0.02 | 0.05 | 0.13 | 0.06 | 0.06 |

Model 1: adjusted for age and gender.

Model 2: adjusted for age, gender, body mass index, the presence of hypertension, dyslipidemia, diabetes mellitus, current smoker and baseline BAD.

BAD indicates brachial artery diameter.

**Supplemental Table S40.** Multiple Linear Regression Analyses of the Relationships between Flow-mediated Vasodilation and Eosinophils in Subjects under 65 Years of Age

| Variable | Unadjusted | | Model 1 | | Model 2 | |
| --- | --- | --- | --- | --- | --- | --- |
|  | β | P value | β | P value | β | P value |
| Eosinophils, × 10^3^/μL | 0.10 | 0.03 | 0.11 | 0.01 | 0.09 | 0.03 |

Model 1: adjusted for age and gender.

Model 2: adjusted for age, gender, body mass index, the presence of hypertension, dyslipidemia, diabetes mellitus, current smoker and baseline BAD.

BAD indicates brachial artery diameter.

**Supplemental Table S41.** Multiple Linear Regression Analyses of the Relationships between Flow-mediated Vasodilation and Lymphocytes in Subjects under 65 Years of Age

| Variable | Unadjusted | | Model 1 | | Model 2 | |
| --- | --- | --- | --- | --- | --- | --- |
|  | β | P value | β | P value | β | P value |
| Lymphocytes, × 10^3^/μL | -0.10 | 0.02 | -0.08 | 0.06 | -0.06 | 0.10 |

Model 1: adjusted for age and gender.

Model 2: adjusted for age, gender, body mass index, the presence of hypertension, dyslipidemia, diabetes mellitus, current smoker and baseline BAD.

BAD indicates brachial artery diameter.

**Supplemental Table S42.** Multiple Linear Regression Analyses of the Relationships between Flow-mediated Vasodilation and Basophils in Subjects with 65 Years of Age or Older

| Variable | Unadjusted | | Model 1 | | Model 2 | |
| --- | --- | --- | --- | --- | --- | --- |
|  | β | P value | β | P value | β | P value |
| Basophils, × 10^3^/μL | 0.10 | 0.02 | 0.09 | 0.03 | 0.10 | 0.01 |

Model 1: adjusted for age and gender.

Model 2: adjusted for age, gender, body mass index, the presence of hypertension, dyslipidemia, diabetes mellitus, current smoker and baseline BAD.

BAD indicates brachial artery diameter.

**Supplemental Table S43.** Multiple Linear Regression Analyses of the Relationships between Nitroglycerine-induced Vasodilation and Lymphocytes in Subjects with 65 Years of Age or Older

| Variable | Unadjusted | | Model 1 | | Model 2 | |
| --- | --- | --- | --- | --- | --- | --- |
|  | β | P value | β | P value | β | P value |
| Lymphocytes, × 10^3^/μL | 0.10 | 0.04 | 0.08 | 0.08 | 0.04 | 0.30 |

Model 1: adjusted for age and gender.

Model 2: adjusted for age, gender, body mass index, the presence of hypertension, dyslipidemia, diabetes mellitus, current smoker and baseline BAD.

BAD indicates brachial artery diameter.

**Supplemental Table S44.** Multiple Linear Regression Analyses of the Relationships between Nitroglycerine-induced Vasodilation and Basophils in Subjects under 35 Years of Age

| Variable | Unadjusted | | Model 1 | | Model 2 | |
| --- | --- | --- | --- | --- | --- | --- |
|  | β | P value | β | P value | β | P value |
| Basophils, × 10^3^/μL | -0.38 | <0.01 | -0.36 | <0.01 | -0.41 | <0.01 |

Model 1: adjusted for age and gender.

Model 2: adjusted for age, gender, body mass index, the presence of hypertension, dyslipidemia, diabetes mellitus, current smoker and baseline BAD.

BAD indicates brachial artery diameter.

**Supplemental Table S45.** Multiple Linear Regression Analyses of the Relationships between Flow-mediated Vasodilation and Eosinophils in Subjects with 55-64 Years of Age

| Variable | Unadjusted | | Model 1 | | Model 2 | |
| --- | --- | --- | --- | --- | --- | --- |
|  | β | P value | β | P value | β | P value |
| Eosinophils, × 10^3^/μL | 0.16 | 0.01 | 0.21 | <0.01 | 0.14 | 0.03 |

Model 1: adjusted for age and gender.

Model 2: adjusted for age, gender, body mass index, the presence of hypertension, dyslipidemia, diabetes mellitus, current smoker and baseline BAD.

BAD indicates brachial artery diameter.

**Supplemental Table S46.** Multiple Linear Regression Analyses of the Relationships between Nitroglycerine-induced Vasodilation and Lymphocytes in Subjects with 65-74 Years of Age

| Variable | Unadjusted | | Model 1 | | Model 2 | |
| --- | --- | --- | --- | --- | --- | --- |
|  | β | P value | β | P value | β | P value |
| Lymphocytes, ×10^3^/μL | 0.20 | <0.01 | 0.15 | <0.01 | 0.08 | 0.12 |

Model 1: adjusted for age and gender.

Model 2: adjusted for age, gender, body mass index, the presence of hypertension, dyslipidemia, diabetes mellitus, current smoker and baseline BAD.

BAD indicates brachial artery diameter.

**Supplemental Table S47.** Multiple Linear Regression Analyses of the Relationships between Nitroglycerine-induced Vasodilation and Monocytes in Subjects with 75 Years of Age and Older

| Variable | Unadjusted | | Model 1 | | Model 2 | |
| --- | --- | --- | --- | --- | --- | --- |
|  | β | P value | β | P value | β | P value |
| Monocytes, × 10^3^/μL | -0.18 | 0.04 | -0.15 | 0.05 | -0.07 | 0.31 |

Model 1: adjusted for age and gender.

Model 2: adjusted for age, gender, body mass index, the presence of hypertension, dyslipidemia, diabetes mellitus, current smoker and baseline BAD.

BAD indicates brachial artery diameter.

**Supplemental Table S48.** Multiple Linear Regression Analyses of the Relationships between Nitroglycerine-induced Vasodilation and Lymphocytes in Subjects with Body Mass Index under 25 kg/m^2^

| Variable | Unadjusted | | Model 1 | | Model 2 | |
| --- | --- | --- | --- | --- | --- | --- |
|  | β | P value | β | P value | β | P value |
| Lymphocytes, × 10^3^/μL | 0.11 | 0.01 | 0.10 | 0.02 | 0.10 | 0.01 |

Model 1: adjusted for age and gender.

Model 2: adjusted for age, gender, body mass index, the presence of hypertension, dyslipidemia, diabetes mellitus, current smoker and baseline BAD.

BAD indicates brachial artery diameter.

**Supplemental Table S49.** Multiple Linear Regression Analyses of the Relationships between Flow-mediated Vasodilation and Basophils in Subjects with Body Mass Index under 25 kg/m^2^

| Variable | Unadjusted | | Model 1 | | Model 2 | |
| --- | --- | --- | --- | --- | --- | --- |
|  | β | P value | β | P value | β | P value |
| Basophils, × 10^3^/μL | 0.09 | 0.02 | 0.07 | 0.08 | 0.08 | 0.10 |

Model 1: adjusted for age and gender.

Model 2: adjusted for age, gender, body mass index, the presence of hypertension, dyslipidemia, diabetes mellitus, current smoker and baseline BAD.

BAD indicates brachial artery diameter.

**Supplemental Table S50.** Multiple Linear Regression Analyses of the Relationships between Nitroglycerine-induced Vasodilation and Lymphocytes in Subjects without Hypertension

| Variable | Unadjusted | | Model 1 | | Model 2 | |
| --- | --- | --- | --- | --- | --- | --- |
|  | β | P value | β | P value | β | P value |
| Lymphocytes, × 10^3^/μL | 0.21 | <0.01 | 0.17 | 0.01 | 0.15 | 0.03 |

Model 1: adjusted for age and gender.

Model 2: adjusted for age, gender, body mass index, the presence of dyslipidemia, diabetes mellitus, current smoker and baseline BAD.

BAD indicates brachial artery diameter.

**Supplemental Table S51.** Multiple Linear Regression Analyses of the Relationships between Nitroglycerine-induced Vasodilation and Eosinophils in Subjects without Hypertension

| Variable | Unadjusted | | Model 1 | | Model 2 | |
| --- | --- | --- | --- | --- | --- | --- |
|  | β | P value | β | P value | β | P value |
| Eosinophils, × 10^3^/μL | 0.16 | 0.02 | 0.14 | 0.05 | 0.04 | 0.54 |

Model 1: adjusted for age and gender.

Model 2: adjusted for age, gender, body mass index, the presence of dyslipidemia, diabetes mellitus, current smoker and baseline BAD.

BAD indicates brachial artery diameter.

**Supplemental Table S52.** Multiple Linear Regression Analyses of the Relationships between Nitroglycerine-induced Vasodilation and Lymphocytes in Subjects without Diabetes Mellitus

| Variable | Unadjusted | | Model 1 | | Model 2 | |
| --- | --- | --- | --- | --- | --- | --- |
|  | β | P value | β | P value | β | P value |
| Lymphocytes, × 10^3^/μL | 0.08 | 0.04 | 0.06 | 0.11 | 0.07 | 0.06 |

Model 1: adjusted for age and gender.

Model 2: adjusted for age, gender, body mass index, the presence of hypertension, dyslipidemia, current smoker and baseline BAD.

BAD indicates brachial artery diameter.

**Supplemental Table S53.** Multiple Linear Regression Analyses of the Relationships between Flow-mediated Vasodilation and Basophils in Subjects with Diabetes Mellitus

| Variable | Unadjusted | | Model 1 | | Model 2 | |
| --- | --- | --- | --- | --- | --- | --- |
|  | β | P value | β | P value | β | P value |
| Basophils, × 10^3^/μL | 0.12 | 0.03 | 0.11 | 0.03 | 0.13 | 0.01 |

Model 1: adjusted for age and gender.

Model 2: adjusted for age, gender, body mass index, the presence of hypertension, dyslipidemia, current smoker and baseline BAD.

BAD indicates brachial artery diameter.

**Supplemental Table S54.** Multiple Linear Regression Analyses of the Relationships between Nitroglycerine-induced Vasodilation and Lymphocytes in Subjects with Middle White Blood Cell Count

| Variable | Unadjusted | | Model 1 | | Model 2 | |
| --- | --- | --- | --- | --- | --- | --- |
|  | β | P value | β | P value | β | P value |
| Lymphocytes, × 10^3^/μL | 0.15 | <0.01 | 0.13 | 0.01 | 0.11 | 0.02 |

Model 1: adjusted for age and gender.

Model 2: adjusted for age, gender, body mass index, the presence of hypertension, dyslipidemia, diabetes mellitus, current smoker and baseline BAD.

BAD indicates brachial artery diameter.

**Supplemental Table S55.** Multiple Linear Regression Analyses of the Relationships between Nitroglycerine-induced Vasodilation and Monocytes in Subjects with Middle White Blood Cell Count

| Variable | Unadjusted | | Model 1 | | Model 2 | |
| --- | --- | --- | --- | --- | --- | --- |
|  | β | P value | β | P value | β | P value |
| Monocytes, × 10^3^/μL | -0.13 | 0.02 | -0.08 | 0.14 | -0.04 | 0.37 |

Model 1: adjusted for age and gender.

Model 2: adjusted for age, gender, body mass index, the presence of hypertension, dyslipidemia, diabetes mellitus, current smoker and baseline BAD.

BAD indicates brachial artery diameter.

**Supplemental Table S56.** Multiple Linear Regression Analyses of the Relationships between Nitroglycerine-induced Vasodilation and Lymphocytes in Subjects with Low baseline BAD

| Variable | Unadjusted | | Model 1 | | Model 2 | |
| --- | --- | --- | --- | --- | --- | --- |
|  | β | P value | β | P value | β | P value |
| Lymphocytes, ×10^3^/μL | 0.12 | 0.01 | 0.08 | 0.08 | 0.08 | 0.06 |

Model 1: adjusted for age and gender.

Model 2: adjusted for age, gender, body mass index, the presence of hypertension, dyslipidemia, diabetes mellitus and current smoker.

BAD indicates brachial artery diameter.

**Supplemental Table S57.** Multiple Linear Regression Analyses of the Relationships between Nitroglycerine-induced Vasodilation and Eosinophils in Subjects with Low baseline BAD

| Variable | Unadjusted | | Model 1 | | Model 2 | |
| --- | --- | --- | --- | --- | --- | --- |
|  | β | P value | β | P value | β | P value |
| Eosinophils, × 10^3^/μL | 0.09 | 0.04 | 0.07 | 0.14 | 0.05 | 0.25 |

Model 1: adjusted for age and gender.

Model 2: adjusted for age, gender, body mass index, the presence of hypertension, dyslipidemia, diabetes mellitus and current smoker.

BAD indicates brachial artery diameter.

**Supplemental Table S58.** Multiple Linear Regression Analyses of the Relationships between Flow-mediated Vasodilation and Eosinophils in Subjects with High baseline BAD

| Variable | Unadjusted | | Model 1 | | Model 2 | |
| --- | --- | --- | --- | --- | --- | --- |
|  | β | P value | β | P value | β | P value |
| Eosinophils, × 10^3^/μL | 0.11 | 0.01 | 0.08 | 0.08 | 0.08 | 0.06 |

Model 1: adjusted for age and gender.

Model 2: adjusted for age, gender, body mass index, the presence of hypertension, dyslipidemia, diabetes mellitus and current smoker.

BAD indicates brachial artery diameter.
